# Enriching Traditional Protein-protein Interaction Networks with Alternative Conformations of Proteins

**DOI:** 10.1038/s41598-017-07351-0

**Published:** 2017-08-03

**Authors:** Farideh Halakou, Emel Sen Kilic, Engin Cukuroglu, Ozlem Keskin, Attila Gursoy

**Affiliations:** 1 Department of Computer Engineering, Koc University, Istanbul, 34450 Turkey; 2 Department of Chemical and Biological Engineering, Koc University, Istanbul, 34450 Turkey; 3 Computational Sciences and Engineering, Graduate School of Sciences and Engineering, Koc University, Istanbul, 34450 Turkey; 4 Microbiology, Immunology and Cell Biology Department, West Virginia University, Morgantown, 26505 WV USA

## Abstract

Traditional Protein-Protein Interaction (PPI) networks, which use a node and edge representation, lack some valuable information about the mechanistic details of biological processes. Mapping protein structures to these PPI networks not only provides structural details of each interaction but also helps us to find the mutual exclusive interactions. Yet it is not a comprehensive representation as it neglects the conformational changes of proteins which may lead to different interactions, functions, and downstream signalling. In this study, we proposed a new representation for structural PPI networks inspecting the alternative conformations of proteins. We performed a large-scale study by creating breast cancer metastasis network and equipped it with different conformers of proteins. Our results showed that although 88% of proteins in our network has at least two structures in Protein Data Bank (PDB), only 22% of them have alternative conformations and the remaining proteins have different regions saved in PDB. However, using even this small set of alternative conformations we observed a considerable increase in our protein docking predictions. Our protein-protein interaction predictions increased from 54% to 76% using the alternative conformations. We also showed the benefits of investigating structural data and alternative conformations of proteins through three case studies.

## Introduction

The most common representation of PPI networks is a graph demonstration. In these PPI graphs, nodes represent the proteins and edges represent their interactions. This abstract representation provides a global picture of biological processes and protein function and helps us to simplify complex cellular systems^1^. However, to deeply understand functional roles and binding mechanisms of proteins, we need to include an extra piece of information in these PPI networks which comes from structural data.

Addition of the structural information to the traditional PPI networks enables us to answer some essential questions in systems biology: (A) The first question would be, how is it possible for some proteins to have tens and even hundreds of interactions in PPI networks? Since proteins have a limited surface area, a single protein cannot interact with such a large number of partners at the same time. Tsai *et al*.^2^ show that the problem arises from the traditional representation of PPI networks. In these networks, all the protein products of a single gene are mapped into a single node. So, in protein interaction networks, each node represents a collection of proteins, each with a distinct conformation or spliced form. Even though the conformations are not totally different, the small differences suffice to bring new interactions. Nevertheless, the number of interactions can vary from one protein to another. Structural analysis suggests that the essential proteins in the PPI networks have more binding sites than other proteins^3^. On the other hand, it has been shown that the larger the number of partners a hub protein has, the higher the probability of the hub essentiality is^4^. (B) The next question would be, which interactions can occur simultaneously and which are mutually excluded? To answer this question, we need to investigate a new dimension in PPI networks which is time^5^. One of the first pioneering works in this field was done by Kim *et al*.^6^. They mapped the protein interactions to known interface structures by using their sequence similarity. They identified mutually exclusive interactions by inspecting the usage of the same binding interface on their structural yeast interaction network. Similar to this study, Tuncbag *et al*.^5^ used p53 pathway as a case study and predicted the simultaneous and mutually exclusive interactions in their network. Kuzu *et al*.^7^ used structural interaction networks to find simultaneous and mutually exclusive interactions on ERK and MAPK pathways. C) Another question would be, how can mutations, located on interfaces, surfaces, or buried in the core regions of proteins, affect the PPI networks? It has been shown that mutations located on interaction interfaces of a protein are more likely causing distinct interruptions in the overall interactome^8^. Therefore, they can result in different biological phenotypes. In a recent study, Mosca *et al*.^9^ created a resource named dSysMap which systematically maps disease-related missense mutations on the structurally annotated human interactome. Their study shows that if pairs of mutations are located in different interfaces of the same protein, they usually cause different phenotypes. However, if they are located in the same interface of interacting proteins, they are most likely producing the same phenotype. To predict the effects of mutations on PPI networks, Moretti *et al*.^10^ performed a community-wide assessment of available methods. They computationally designed influenza binders HB36.4 and HB80.3, and then created single point mutant variants corresponding to all 20 amino acids at each position of the binders. They showed that mutations can influence binding if they disrupt the folded state.

**Figure 1:**
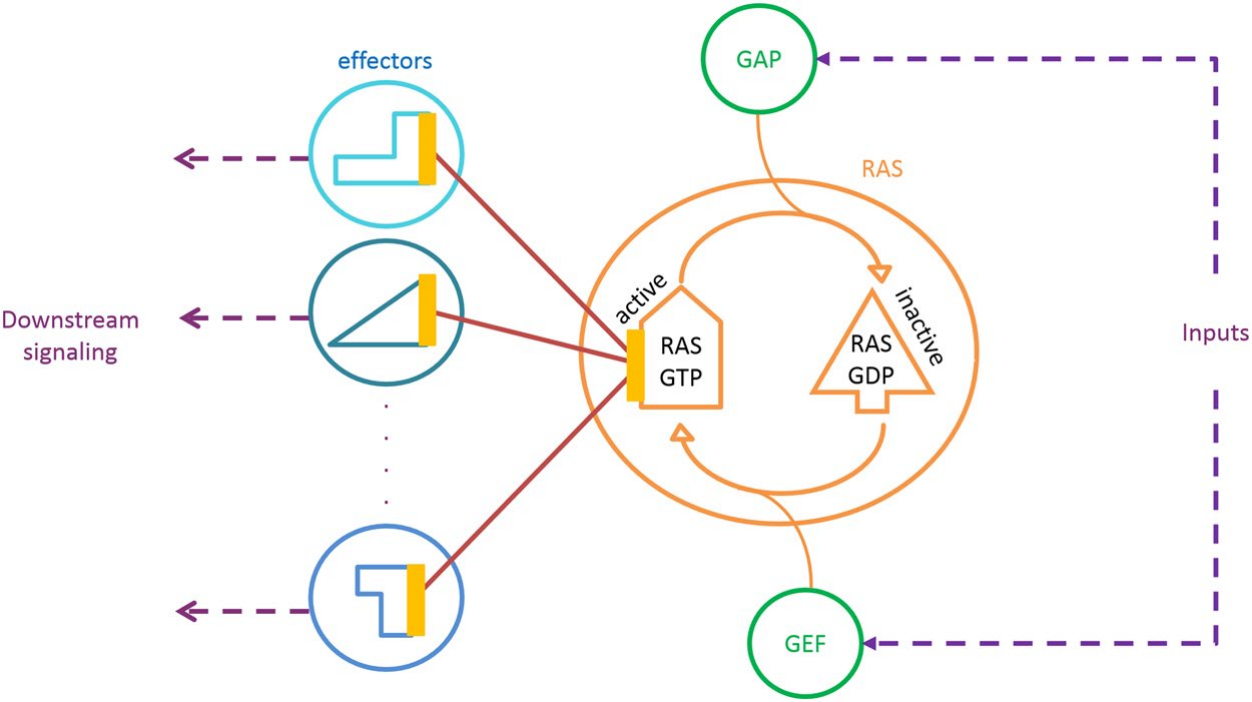
Schematic representation of RAS protein conformational states. In the active form, RAS can bind to its effectors and activate different signalling pathways.

The common practice in structural PPI networks is to inspect just one protein structure for each node. However, proteins are flexible^11, 12^ and their conformations change based on the factors like post-translational modifications, pH level, and environment. Thus, we need to consider an ensemble of protein conformers for each node in PPI networks. The conformational changes dictate the protein function. Therefore, characterization of this association between protein conformation and its function, helps us to understand how to alter and regulate protein activity. The importance of investigating multiple conformers of the proteins in PPI networks lies in the fact that protein conformational changes cause activation of specific pathways^13–15^. A proper example could be RAS-family proteins. RAS proteins switch between GDP-bound inactive form to the GTP-bound active form, as two conformational states, in response to receptor-mediated extracellular signals^16^. They regulate proliferation, differentiation, motility and cytoskeletal re-organization^17, 18^. The conversion of GTP-bound to GDP-bound form is regulated by guanine nucleotide exchange factors (GEFs), and the conversion of GDP-bound to GTP-bound form is mediated by GTPase activating proteins (GAPs)^19^. Active RAS interacts with its effector proteins and activates downstream effectors like Raf kinase, phosphatidylinositol 3-kinase and Ral guanine nucleotide-dissociation stimulator^20^. Schematic representation of RAS conformational states and their corresponding interactions are shown in Fig. 1.

RAS example intensifies the investigation of different conformers of proteins in structural PPI networks. Protein Data Bank (PDB)^21^ provides protein structures which include alternative conformations of the same protein coming from different experiments. Kuzu *et al*.^22^ used an ensemble of protein conformations to dock the difficult cases of a docking benchmark data set. Applying the alternative conformations of proteins significantly increased the probability of successful protein interaction prediction in their study. They created the network of ERK interactions as a case study. Also, ensemble of protein conformations are used in docking to model small-molecule-protein or peptide-protein interactions^23, 24^. However, ensemble docking studies are neither at network level, nor for protein interactions, they usually make use of computationally generated conformations rather than multiple conformations obtained from PDB.

There are some recent databases which explore the conformational changes and flexibility of protein structures deposited in PDB database such as CCProf^25^, PDBFlex^26^, and CoDNaS 2.0^27^. PDBFlex database provides the analysis of structural variations between the different depositions for the same protein in the PDB database. It clusters different structures of proteins based on their sequence similarity and animates its dynamics. CCProf database also offers conformational changes of protein structures plus some other beneficial biological features like potential binding target site, secondary structure, conservation, phosphorylation site and catalytic site. CoDNaS database covers a large proportion of available protein structures derived from X-ray crystallography and NMR experiments in the PDB database. It clusters the protein structures using their sequence identity too. There are ~15 conformers on average for each protein chain in this database.

In this study, we concentrate on mapping structural data into the PPI networks and investigating alternative conformations of these protein structures. We have chosen the breast cancer lung and brain metastasis PPI networks of our previous study^28^. After creating the traditional PPI network of breast cancer lung and brain metastasis, we enriched them by alternative conformations of the proteins. This new enriched network helps us to understand how proteins interact with each other using specific conformations and how these alternative conformations lead to diverse downstream signalling. It also shows us the effects of mutations on protein conformation, its interaction and function.

## Materials and Methods

**Figure 2:**
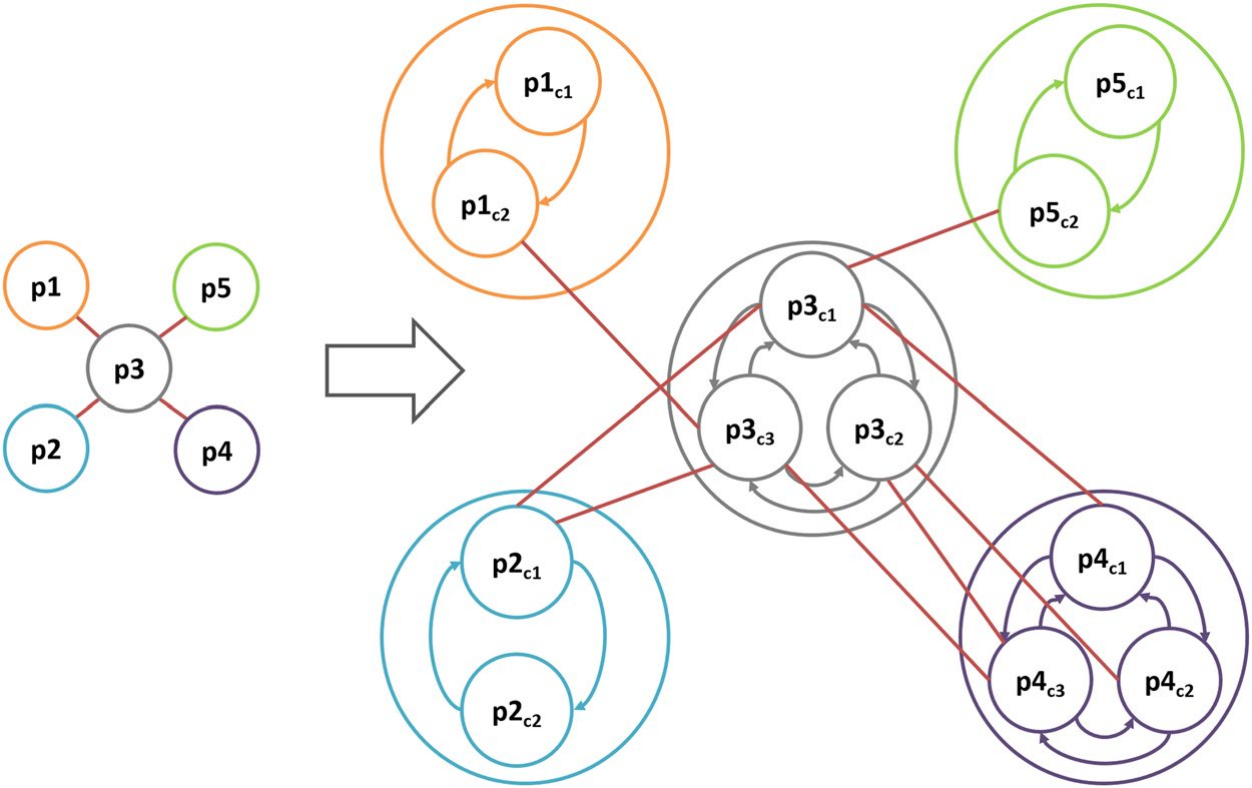
Structural PPI network in traditional representation on the left, and the new representation investigating proteins conformational changes on the right. Arrows show inter-conversions between alternative conformations of the same protein.

To have a more realistic view of PPI networks, we propose a new PPI representation as in Fig. 2. This representation covers proteins’ alternative conformations and shows their specific interactions with their binding partners. In this representation, each protein p in the network is an ensemble of that proteins states/conformations {p_c1_, p_c2_, … p_cn_} which can inter-convert to each other. If p1 and p2 have N and M alternative conformations respectively, the edge {p1, p2} shows that at least one {p1_ci_, p2_cj_} i ≤ N, j ≤ M is physicochemically possible. This new representation reveals two important facts about PPI networks. First, each specific protein conformer can contribute to a number of potential interactions the protein can have. In Fig. 2, for example, if p3 is in conformation 2 (p3_c2_), it can only bind to p4 if p4 is in its c2 or c3 conformations. However, if p3 is in conformation c3, it may bind to p1, p2, or p3. Second, each edge in the PPI network has a strength based on the present conformations of the proteins in the network at any time point. Namely, some edges can be formed more probably as the corresponding interacting proteins can bind to each other using different conformations. For example, edge {p3, p5} can be speculated to be less probable than {p3, p4}, as the latter can happen regardless of which conformation p3 has.

**Figure 3:**
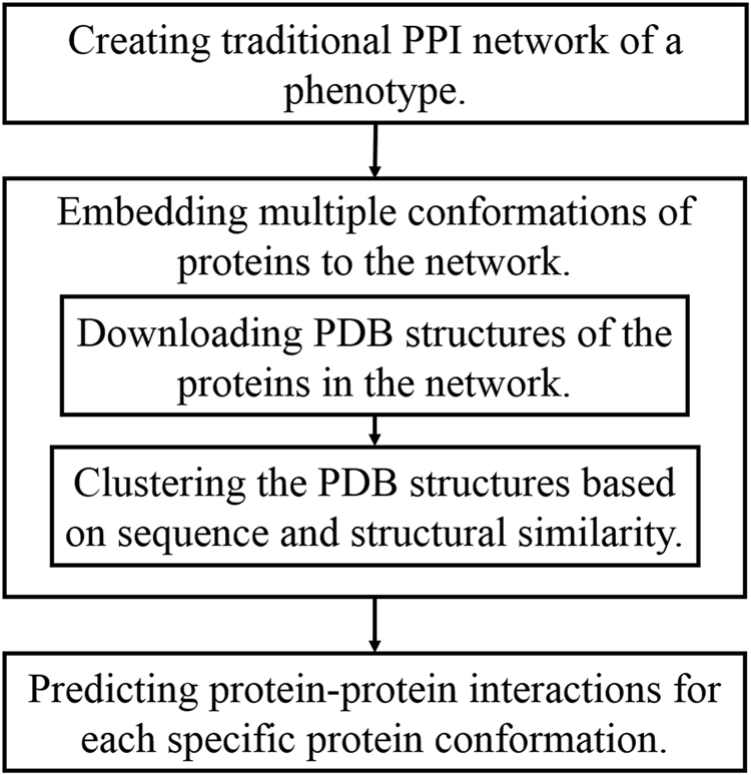
Flowchart of creating the multi-conformational PPI network.

**Figure 4:**
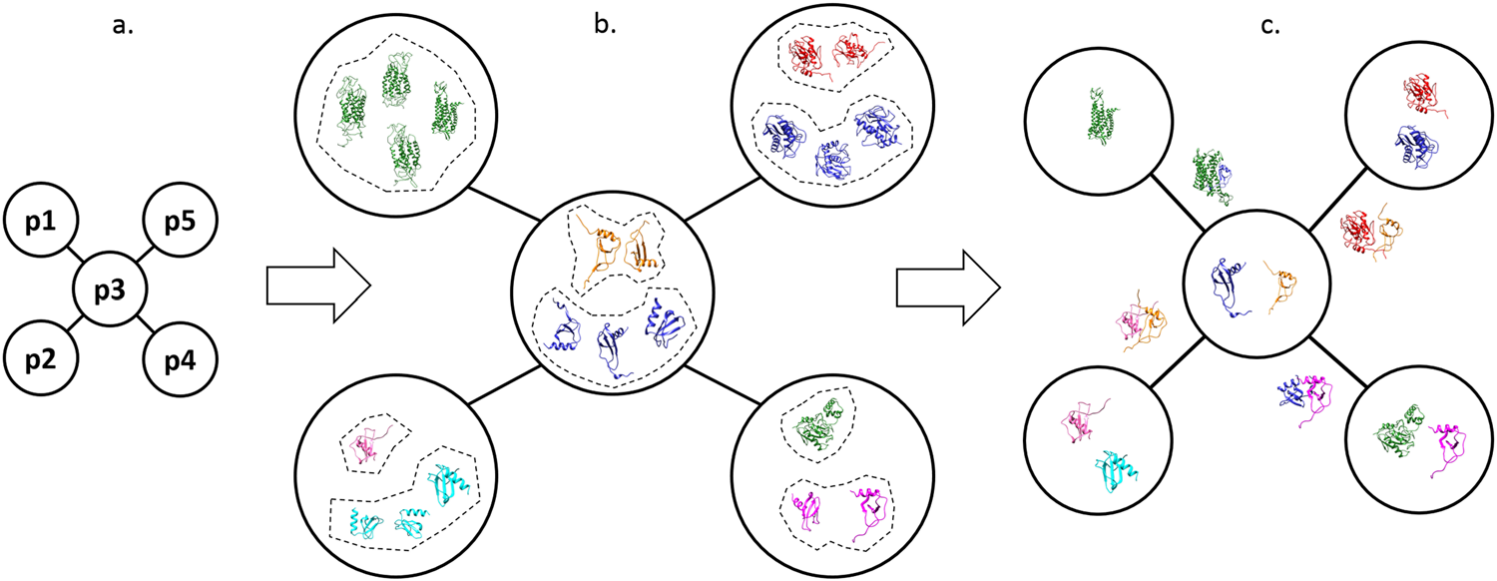
Graphical illustration of creating the multi-conformational PPI network. (**a**) Traditional PPI network. Each node represents a protein. (**b**) Available PDB structures for each protein. Similar conformations for each protein, indicated with the same colour, are clustered together. (**c**) For each cluster, just one PDB structure is selected as its representative. Two proteins may bind to each other by using specific conformations.

To create the multi-conformational PPI network, we followed the steps shown in Fig. 3. The process starts with creating a traditional PPI network for the phenotype of interest. In this network, nodes show the proteins and edges show the protein-protein interactions. Afterwards, available PDB structures of each protein in the network are mapped to it. Protein structures are all stored in PDB database^21^. As it is a redundant database, mapping all of the structures without any curation can cause unnecessary repetition in the 3D protein-protein interaction predictions. Thus, similar PDB structures which correspond to the same protein should be eliminated. For each protein, we cluster its alternative conformations according to their sequence and structural similarities. Then, the possible protein-protein interactions using each specific protein conformation are investigated using a docking method. We use PRISM in this study for interaction prediction because unlike many docking methods designed just for a binary interaction, PRISM is able to get a PPI network as an input which makes it suitable for large scale studies. The graphical illustration of the multi-conformational PPI network creation procedure is illustrated in Fig. 4.

After clustering the protein structures according to their similarities, one or more structures remain as their clusters’ representatives for each protein. To predict if two connected proteins in the network can bind to each other or not, we explored all permutations of their representative structures. As we can see in the results section, some interactions in the network are feasible only by using specific conformations of the interacting proteins. This fact may change the topology of the network at each specific time point as each protein can form different interactions based on its conformation in the network. This is one of the major points that traditional PPI networks are not able to show.

### Clustering PDB Structures to Form Alternative Conformations

We cluster protein structures using their sequence identity and structural similarity, and choose a representative structure for each cluster representing an alternative conformation of the same protein in our network. First, we use UniProt Knowledgebase^29^ to map proteins to their known 3D structures which provides a list of corresponding available structures in PDB. In this step, protein structures which have less than 30 residues are eliminated. Secondly, all available structures for each UniProt ID are structurally aligned using TMAlign tool^30^ to each other to quantify their structural differences. In order to find the unique protein structures, we use agglomerative complete linkage clustering method based on sequence similarity and RMSD value of the protein structures. If two protein structures, corresponding to the same UniProt ID, have more than 95% sequence identity and smaller than 2 Å RMSD value, we put them together in a cluster. We applied this procedure until there is not any protein structures left without an assigned cluster information. Representative structures for each cluster are selected based on the best intra-cluster similarity and RMSD value. These representative structures are the alternative structures of a node corresponding to a protein in our merged structural network (Supplementary Data 3). We should note that it is possible that some clusters represent different parts of the same protein because PDB structures do not necessarily cover the complete protein sequence. Since they all correspond to different parts of the protein, they still will contribute to some specific interactions in the PPI network. Therefore, we keep all representatives in the network.

### Creating Breast Cancer Metastases Network

Advances in systems biology over the past years have provided a large amount of experimental high-throughput data on protein interactions. This information is spread across multiple databases with some overlap. In addition, given the variations in the experimental techniques used, each database has a different level of reliability for its data. Therefore, a tool is required to unify these data independently of the identifiers used in each database. We constructed the PPI network for breast cancer lung and brain metastasis by using GUILDify webserver^31^. This webserver uses BIANA^32^ to construct the network and scores the nodes by using GUILD^33^, which is based on network-based prioritization algorithms. GUILDify webserver first maps the seed genes, which are previously known to be associated with a specific phenotype, into a genome wide human interaction network provided by BIANA and scores other genes based on their association with the seed genes by using a combination of network-based parameters i.e. NetScore, NetZscore and NetShort^32^.

We made use of the genes identified by Minn *et al*.^34^ and Bos *et al*.^35^ as seed genes, which were also used previously by Engin *et al*.^36^ as well, to generate lung and brain metastasis subnetworks of breast cancer. They identified 18 genes mediating breast cancer to lung metastasis and 17 genes mediating breast cancer to brain metastasis. Supplementary Table S1 shows the seed genes used. GUILDify scores genes based on their associations with brain and lung metastasis phenotypes. Thus, each node (gene) has two separate scores which lead to two different subnetworks namely BMSN (Brain Metastasis SubNetwork) and LMSN (Lung Metastasis SubNetwork). BMSN and LMSN can be found in Supplementary Data 1 and 2 files. The GUILDify scores were presented only for the genes but not for their interactions (edges). So we calculated the score of the interactions by averaging the scores of their associated genes. The score of each interaction shows its relevance to the seed genes. GUILDify score varies between 0 and 1, so as the interaction scores. The score close to 1 indicates stronger relevance to a specific phenotype.

We merged the two subnetworks i.e. BMSN and LMSN, produced by GUILDify webserver to have a single network. This network consisted of 12,172 genes and 324,280 interactions coming from various databases including UniProt^29^, TREMBL^37^, GO^38^, OMIM^39^, Reactome^40^, INTACT^41^, MINT^42^, DIP^43^, and HPRD^44^.

### Filtering the Network

The examination of the interacting protein pairs and their affinities from experimental methods includes both direct and indirect interactions. As we aimed to use only physical protein interactions, we filtered our data by using experimental protein interactions from STRING database^45^ with medium confidence score to eliminate bulk network clusters and further computational load in 3D protein interaction analysis. STRING database collects available experimental information coming from primary interaction databases and assigns confidence score by considering both direct and indirect interactions. Using medium confidence score, we aimed to eliminate the PPIs with lower probability to be physical (or direct) interactions.

Using the STRING database^45^, we downloaded Human Interaction Network (HIN) consisting of 165,184 protein interactions. From this network, we only considered protein interactions coming from experimental data with the confidence score ≥0.4 (Supplementary Table S2).

After creation of the STRING human interaction network, we compared it with our merged network and selected the interactions presented in both networks. After this process, our network diminished to 43,903 interactions and 9,544 genes. Before filtering the network, there were many proteins with extremely high degrees. As an example, FSCN1 had 804 neighbours and it had a mesh-like cluster. However, after filtering the network its degree decreased to only 18 nodes.

To proceed with structural analysis, we selected the first 1000 interactions having highest GUILDify scores from each subnetwork. GULDify scores of edges range between 0.08–0.55 and 0.04–0.58 in BMSN and LMSN respectively. There were 95 nodes and 73 edges in common between BMSN and LMSN (Supplementary Fig. S1). The merged network contained 1927 interactions as shown in Table 1, where 1240 of them have experimental evidence for binding, 497 of these 1240 interactions are physical interactions and 107 of them come from APMS.

**Table 1:** Coverages of PDB and PRISM in our subnetworks.

Network	Original Network		Covered by PDB		Covered by PRISM	
	#of nodes	#of edges	#of nodes	#of edges^a^	#of edges^b^ using single conformation	#of edges using multiple conformations
BMSN	567	1000	262	376	186	254
LMSN	622	1000	280	387	224	322
Merged Network	1094	1927	484	720	390	547

^a^If proteins at both ends of an edge have PDB structures.

^b^If PRISM has a prediction for that edge.

We used Cytoscape^46^ to visualize the subnetworks. Topological and functional analyses of the subnetworks can be found in our previous study^28^.

## Results and Discussion

### Mapping Protein Conformations to Network

**Figure 5:**
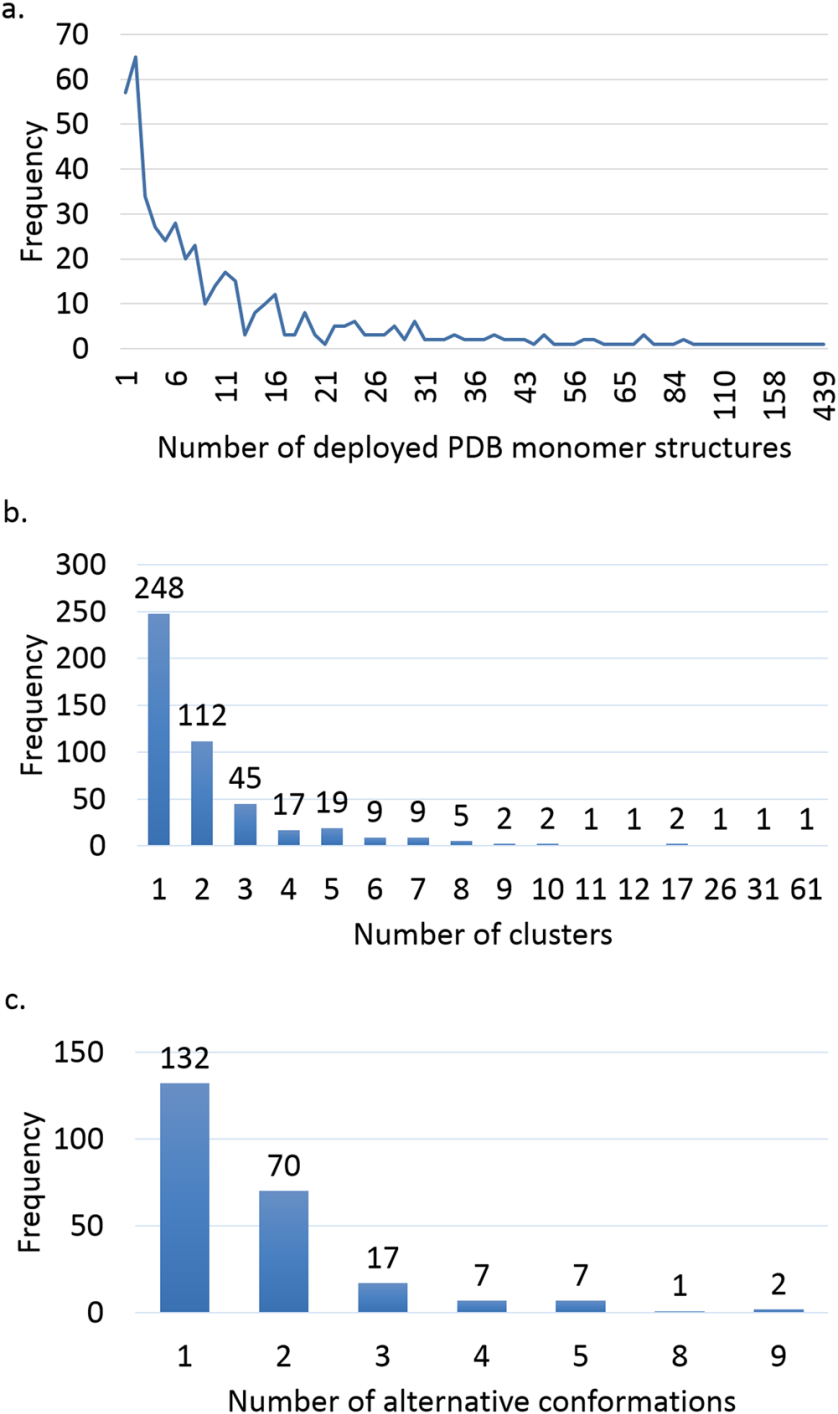
PDB database and clustering statistics. (**a**) Number of deployed monomer structures for each protein in the PDB database. (**b**) Number of different clusters for each protein based on our clustering algorithm. These clusters may represent different parts of the same protein or alternative conformations of it. (**c**) Number of the alternative conformations each protein has in our network.

We used UniProt Knowledgebase^29^ to map the proteins to their known 3D structures. We cluster the protein structures corresponding to the same UniProt ID according to their sequence identity and structural alignment using the clustering algorithm described in Materials and Methods section. Figure 5 shows the clustering statistics of the proteins in our subnetworks. Figure 5a represents the number of monomer structures deployed for each protein in the PDB database. As can be seen from the figure, ~90% of our network’s proteins have at least two available monomer structures in the PDB database. HBA1 (hemoglobin subunit alpha 1) is the protein with maximum number of available structures in our network with 439 PDB monomer structures. Figure 5b shows the number of clusters for each protein based on our clustering algorithm. As can be seen from the figure, ~50% of the proteins has just one cluster meaning that all their PDB structures shows the same conformation of the protein. After clustering, HBA1’s PDB structures grouped in just 3 different clusters. In this step, APP (amyloid beta precursor protein), with 237 PDB monomer structures, had the maximum number of clusters with 61 clusters. As we mentioned earlier, PDB structures of a protein may cover different parts of the protein. So, if a protein has 3 clusters based on our clustering algorithm, it does not necessarily mean that it has three alternative conformations in the PDB database as those clusters may represent different overlapping/non-overlapping parts of the protein. Two clusters are considered overlapping if they share two or more residues. Therefore, to discover the exact number of alternative conformations each protein has, we mined the protein clusters, for the proteins having at least two clusters, to find the different PDB structures covering the same part of the protein. Figure 5c shows that ~22% of the proteins in our network has at least two alternative conformations saved in the PDB database. So, the remaining 28% of proteins having at least two clusters, in fact have different regions of proteins saved in PDB database instead of alternative conformations. APP (amyloid precursor protein) and CALM1 (calmodulin 1) have the maximum number of conformations with nine alternative conformations in our network. CALM has 163 PDB monomer structures grouped in 31 clusters.

**Figure 6:**
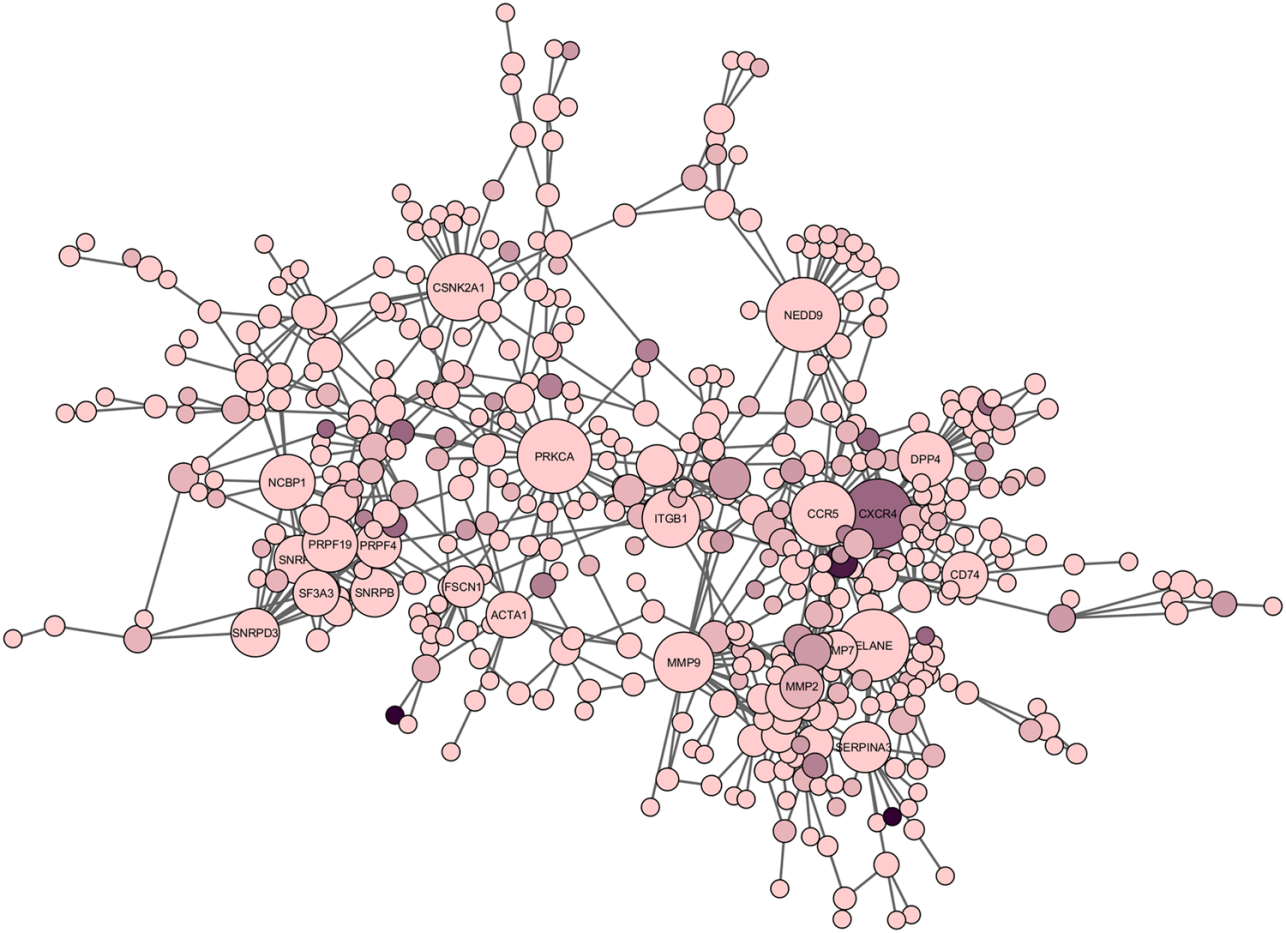
Merged structural network. Node size shows the node degree. Node colour corresponds to the number of alternative conformations, i.e. the darker the node colour is, the more alternative conformation it has.

Figure 6 shows our merged structural network in which node size represents the node degree in the network and the colour of nodes correspond to alternative conformations, i.e. the darker the colour is, the more alternative conformation it has. As can be seen from the figure, high degree nodes in the network are all light pink except CXCR4 (C-X-C chemokine receptor type 4) which is a small structure consisting of a random coil. Although this observation may seem counterintuitive, it can be explained as follows: our definition of alternative conformation considers large conformational changes for the structures belonging to the same part of a protein, however small local conformational changes in proteins’ binding sites may disrupt a specific interaction or promote another interaction^47^.

### Modelling 3D Structures of the Binary Protein Interactions

We used PRISM webserver^48–50^ for modelling the binary protein interactions in the structural BMSN and LMSN. PRISM uses a template interface set to predict binding sites of two interacting proteins. Its prediction algorithm investigates the surface regions of two proteins and if they are similar to two sides of a crystal or NMR interface structure, it models their complex structure using that template interface. The rationale of using interfaces instead of whole protein structure for docking purposes in PRISM is that similar binding sites can be found even in globally different protein structures^51^. PRISM algorithm consists of four steps. In the first step, it extracts the surfaces of target proteins. Then, it splits the template interfaces into two sides and using MultiProt^52^ it calculates the similarity of each template interface side to the target protein surfaces in the second step. Multiprot can find the structural similarities of proteins in an order-independent manner which is necessary for surface/interface comparisons. After finding two target protein surfaces which are similar to two sides of a template interface, and making sure that at least one hotspot at each side is conserved, PRISM transforms the surfaces to the template interface to form a protein complex in the third step. Finally, in the last step, FiberDock^53, 54^ is used for flexible refinement of the complexes and ranking them based on the binding energy scores. Fiberdock models both backbone and side-chain flexibility. The current PRISM webserver uses 22,604 template interfaces which were extracted from binary protein interactions stored in the PDB database^55^.

Not all the proteins in our subnetworks have available 3D structures in the PDB database, so we could not model their interactions using the PRISM webserver. As Table 1 shows, only ~44% of the proteins in our network have PDB structures. After clustering the protein structures, we investigate all alternative conformations of the interacting proteins for each interaction/edge in our network to predict if they can bind to each other and how they bind. To do so, we submitted more than 4500 PPIs to PRISM. PRISM webserver modelled ~76% of the structural interactions submitted to it (Table 1). To show the effect of using multiple conformations of proteins in PPI predictions instead of a single conformation, we randomly selected one conformation for each of the proteins in our network and look at their PPI predictions. As shown in Table 1, investigating just one conformation for each protein in the network diminished the PRISM predictions by ~22%, so the PRISM coverage of the network edges reduced to just ~54%. Therefore, using one conformation for protein docking is not enough and may bring incomplete results. All the data are available through http://prism.ccbb.ku.edu.tr/data/fr1.

Equipped with protein structures and their conformations in our network, we show the importance of investigating protein structures, their conformational changes, and effects of mutations on the protein-protein interactions through the following three case studies.

### Conformational Selection Mechanism of KPNB1

KPNB1 (Importin subunit beta-1) is a member of Karyopherin family which transports proteins into and out of the nucleus. It has been known that unlike importin α, members of importin β family can bind to proteins directly and import them independently^56^. KPNB1 has 62 interactions in our network in which 41 of its neighbours has 3D structures available in the PDB database as shown in Supplementary Fig. S2.

**Figure 7:**
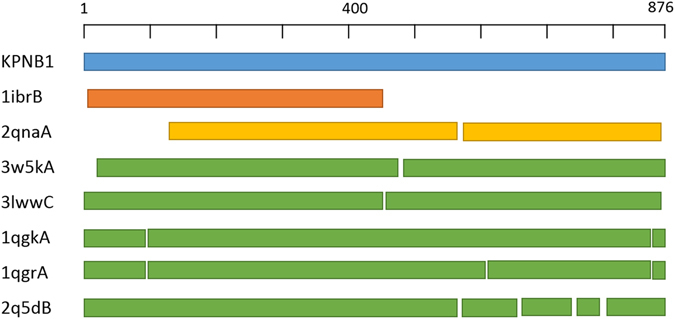
KPNB1 protein and its alternative conformations representative structures after clustering all PDB structures of it.

KPNB1 is a protein of length 876 and there is almost complete structure in the PDB database. There are 19 PDB structures for KPNB1 which are clustered to 7 different clusters based on our algorithm i.e. 3w5kA, 1qgrA, 3lwwC, 1qgkA, 1ibrB, 2q5dB, and 2qnaA. Figure 7 shows a coarse-grained representation of KPNB1 and its 7 different cluster representatives based on our algorithm. As can be clearly seen from the figure, PDB structures 1ibrB and 2qnaA cover different parts of KPNB1 so we don’t use them as alternative conformations of KPNB1 in our analysis. 3D structures of the five alternative conformations of KPNB1, i.e. 3w5kA, 3lwwC, 1qgkA, 1qgrA, and 2q5dB, are shown in Supplementary Fig. S3 and the RMSD values of their aligned structures are shown in Supplementary Table S3. These structures show the open and close conformations of KPNB1.

**Figure 8:**
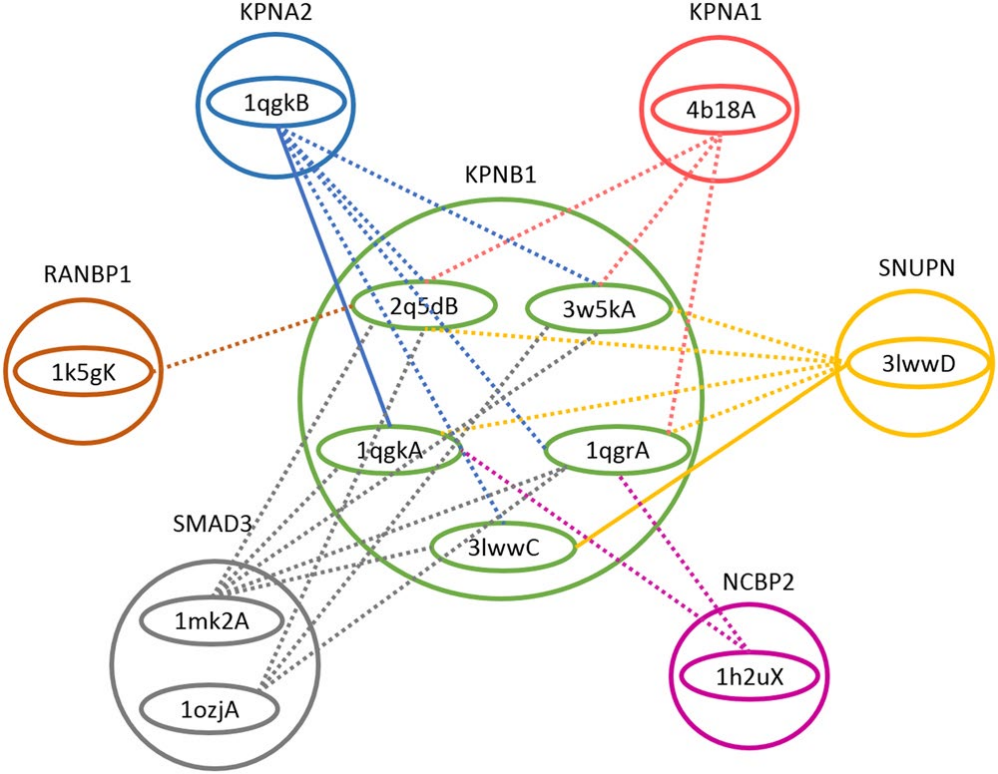
KPNB1’s existing and predicted interactions by PRISM. Interactions having the complex structures in PDB database are shown with solid lines. Dotted lines indicate the PRISM predictions.

We submitted all possible interactions of KPNB1 with its neighbours using their alternative conformations to PRISM. For representation purposes we focus only on 6 different interactions of KPNB1 in our network. Based on the PRISM predictions, we reach the structural network shown in Fig. 8.

**Figure 9:**
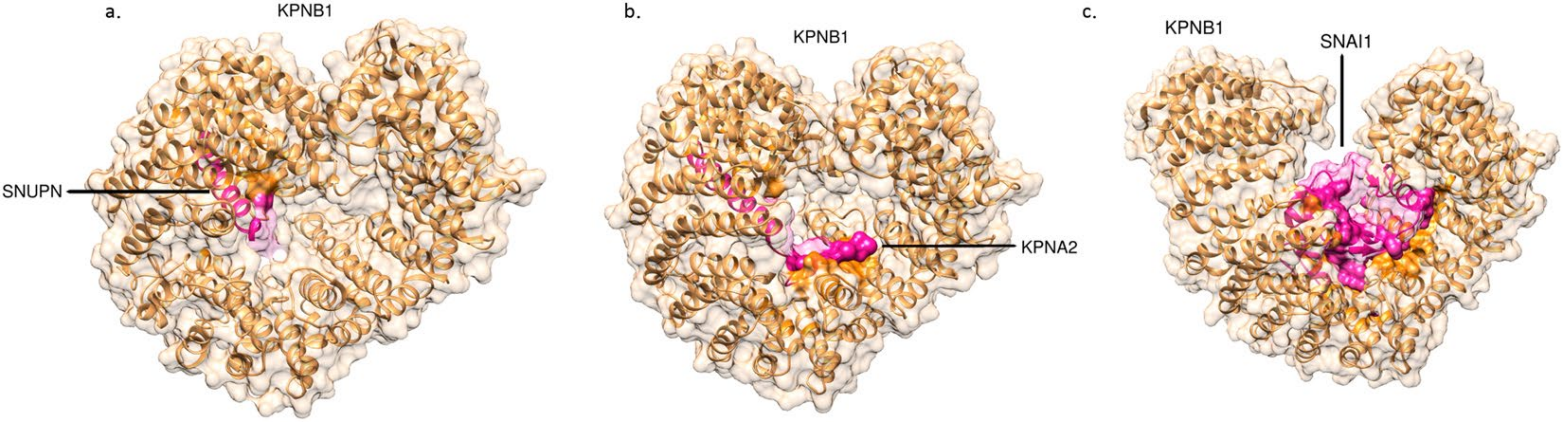
KPNB1 complex structures with. (**a**) SNUPN (PDB ID 3lwwCD) (**b**) KPNA2 (1qgkAB), and (**c**) SNAI1 (3w5kAB). The binding residues on the surface are indicated as opaque.

The complex structures of interactions KPNB1-SNUPN and KPNB1-KPNA2 are already available in the PDB database namely PDB ids 3lww and 1qgk, respectively. The close conformation of KPNB1 i.e. 3lwwC is bound to SNUPN (Snurportin-1). KPNB1 uses SNUPN adapter to bind cargo molecules to import m_3_G-capped U snRNPs^57^. SNUPN has an IBB (importin-β-binding) domain in its N-terminal which is used to bind to KPNB1 and then transport to the nucleus. It has been shown that after each round of import, SNUPN uses CRM1 to return back to the cytoplasm^58^. KPNB1 close conformation 3lwwC bound to SNUPN is represented in Fig. 9a. This complex protein structure has the global binding energy of −49.82 based on the Fiberdock webserver^53, 54^. To detect the binding residues in this complex structure we used HotRegion webserver^59^. The binding residues of this complex structure are listed in Supplementary Table S4, and they are indicated with opaque colours in the figure. We used UCSF Chimera package^60^ for molecular graphics and analyses.

Notwithstanding the high RMSD values between open and close conformations of KPNB1, Bhardwaj and Cingolani^61^ provided structural evidence that open and close conformations of KPNB1 bind to SNUPN IBB domain. We had the same results in PRISM predictions as shown in Fig. 8. It should be noted that, though SNUPN binds to all conformers of KPNB1, it has a different affinity, in terms of binding energy, towards each conformer. For example, the predicted binding energy scores for the interaction of SNUPN with 2q5dB and 1qgkA conformers are −67.38 and −53.32 respectively.

Another existent interaction of KPNB1 is with KPNA2 (PDB id 1qgk). KPNA2 (Importin subunit alpha-1) is an adapter protein for KPNB1 which binds to substrates containing a nuclear localization signal (NLS) motif. Importin β binds to the IBB domain of importin α and forms a heterodimer. Cytosolic proteins having NLS-containing motifs form a complex with this heterodimer and transported into the nucleus by using nuclear pore complexes (NPCs) which are embedded on the nuclear membrane^62, 63^. The available PDB complex structure for KPNB1-KPNA2 uses a KPNB1 close conformer as shown in Fig. 9b. The binding residues of this complex structure are listed in Supplementary Table S5, and they are indicated with opaque colours in the figure. There are 17 residues in common between KPNB1 interacting surface with SNUPN and KPNA2 based on HotRegion webserver^59^ shown with boldface in Supplementary Tables S4 and S5 which indicates that KPNB1 uses almost the same region to bind to these proteins. This complex protein structure has the global binding energy of −138.40 based on the Fiberdock webserver. However, based on the PRISM results, KPNA2 can bind to other conformers of KPNB1 though with significantly fluctuating binding energies e.g. −78.25 and −22.32 to bind to 1qgrA and 3lwwC respectively.

**Figure 10:**
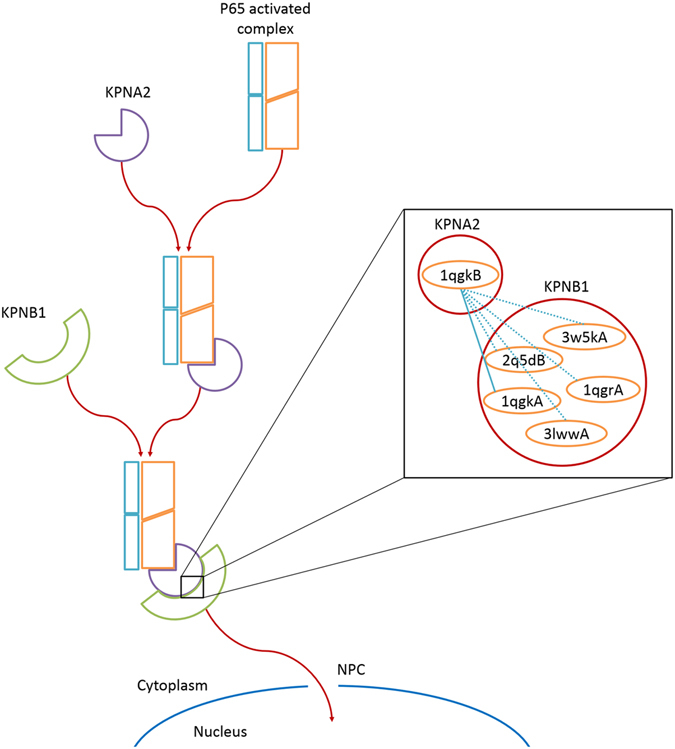
Pathway for p65 import based on Liang *et al*.^64^.

Liang *et al*.^64^ showed that KPNA2/KPNB1 canonical pathway is responsible for p65 (RELA proto-oncogene, NF-kB subunit) import to the nucleus. They tested 6 different members of the importin α family and found that KPNA2 plays the major role in p65 import. p65 uses different members of importin β and α families for its import but it has a high affinity towards KPNB1 and KPNA2. Based on their results, p65 hetero trimer is imported to the nucleus through Nuclear Pore Complexes (NPCs). The proteins involved in this pathway are connected to each other in our network, in the order RELA-KPNA2-KPNB1-NUP153. Figure 10 shows this pathway in which the interaction KPNB1-KPNA2 is represented with its structural details. Despite the large conformational changes of KPNB1, its binding site to KPNA2 remains almost intact enabling it to bind to KPNA2 in different conformations. It is important to know that using which alternative conformations, the proteins involved in a pathway can bind to each other and pass the signal.

By looking at the PDB database, we found that there are other complexes for KPNB1 which complex with RAN, SNAI1, and PTHLH. Among all available structures of KPNB1, only 3w5kA is bound to the zinc finger protein SNAI1. SNAI1 is a transcription factor which has been found in a number of carcinomas and melanomas^65^. Its expression in breast carcinoma is associated with tumour growth and metastasis^66–68^. Besides, *in vivo* experiments showed that silencing of SNAI1 significantly diminishes tumour occurrence and growth^69^.

We would like to know if other conformers of KPNB1 can bind to SNAI1 too, so we submit all other alternative conformations of KPNB1 with SNAI1 to the PRISM webserver. Interestingly, PRISM can only find the complex structure using 2q5dB conformer of KPNB1 for these submissions. In this complex, SNAI1 binds to 2q5dB from the same binding site it uses to interact with 3w5kA. The binding energy score for this complex is −139.17, and the binding residues are listed in Supplementary Data 26. Therefore, KPNB1 conformers 3w5kA and 2q5dB can bind to SNAI1 and import it into the nucleus. KPNB1 open conformation 3w5kA bound to SNAI1 is shown in Fig. 9c. The binding residues of this complex are listed in Supplementary Table S6, and they are indicated with opaque colours in the figure. There are 19 residues in common between KPNB1 interacting surface with SNAI1 and KPNA2 based on Hotregion webserver^59^ shown with italic typeface in Supplementary Tables S5 and S6 which indicates that KPNB1 uses almost the same region to bind to these proteins.

These evidences lead us to infer that some protein conformers are more limited in terms of their binding partners’ quantities e.g. KPNB1 can bind to SNUPN or SNAI1 in open conformation though it can’t bind to SNAI1 in close conformation. Therefore, each specific protein conformer put a limitation on the diversity of possible binding partners.

### Conformational Changes of CXCL12 Leading to Different Downstream Signalling

CXCL12 (C-X-C motif chemokine 12) is the ligand of CXCR4 (C-X-C chemokine receptor type 4) which is a seed gene in LMSN. This interaction has GUILDify score of ~0.5 which places it in the top 5% interactions in LMSN. It is known that *in-vivo* inhibition of these two genes reduce breast cancer metastasis progress of lung^70^. Chemokines are small proinflammatory chemoattractant cytokines which bind to specific G-protein-coupled receptors. CXCL12 is expressed in several organs including lung, liver, brain, skeletal muscle, kidney, heart, skin, and bone marrow. The binding of CXCL12 to CXCR4 is known to induce intracellular signalling through several different pathways initiating signals related to chemotaxis, cell survival and/or proliferation^71^.

The CXCL12-CXCR4 interaction is involved in tumour progression, angiogenesis, metastasis, and survival. There are efforts to block metastatic dissemination by inhibiting CXCR4 activation^72^ to inhibit cancer malignancy^73, 74^. It has been shown that binding to CXCR4 N terminus (CXCR4 1–38) promotes CXCL12 dimerization^75^. Drury *et al*.^76^ found that oligomeric changes of CXCL12 induces cellular migration with monomer but not dimer^77^. Interestingly, they also observed that dimeric CXCL12 exhibited receptor interactions and downstream signalling different from the monomeric chemokine. Their results show that monomeric CXCL12 activates β-arrestin-2 recruitment and filamentous-actin accumulation. On the other hand, dimeric CXCL12 weakly recruits β-arrestin-2 and diminishes actin polymerization compared to monomeric CXCL12^78^. This indicates that monomeric and dimeric CXCL12 can have different outcomes in metastasis phenotypes upon CXCR4 binding.

**Figure 11:**
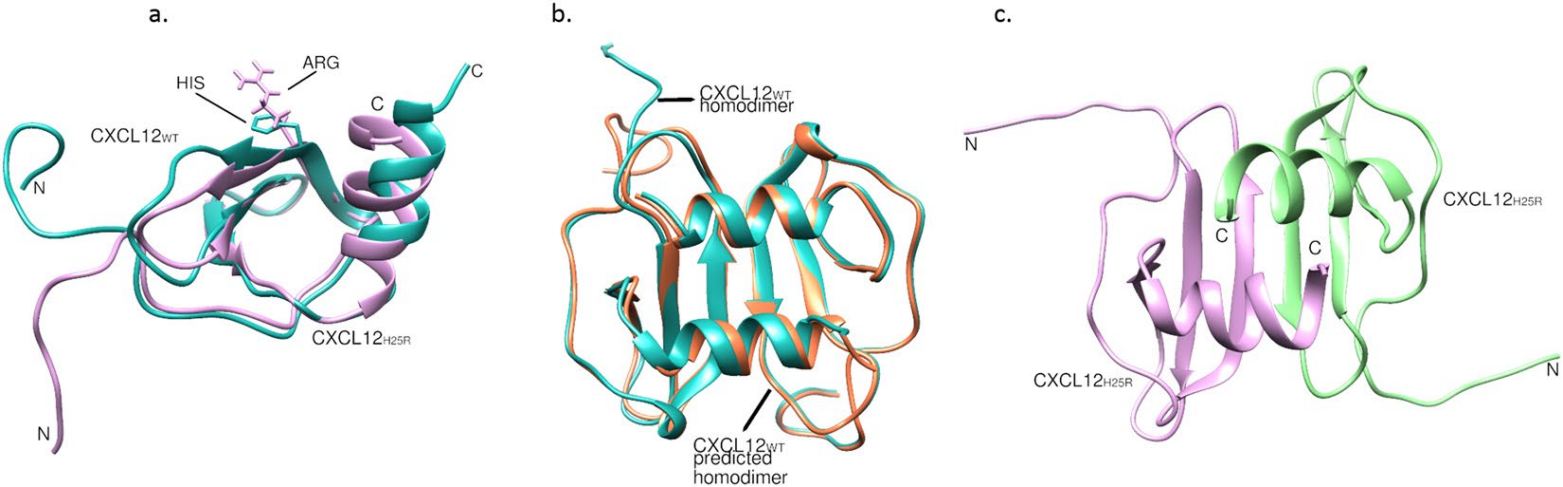
CXCL12 monomer and dimer structures. (**a**) Superposition of CXCL12_WT_ (PDB ID: 2j7zA) and CXL12_H25R_ (PDB ID: 2kolA) conformations. The RMSD value of aligned structures is 6.29 Å. (**b**) Superposition of the PRISM prediction for CXCL12_WT_ homodimer (orange) and the available structure in PDB i.e. 2j7z (blue). (**c**) PRISM prediction for CXCL12_H25R_ homodimer.

Different signalling outcomes might be related to alternative conformations of CXCL12 upon CXCR4 binding. There are 20 available structures in PDB database for CXCL12 which are divided to 11 clusters based on our clustering method. It is known that conformational changes can be related to point mutations on the protein interfaces which may change their dimerization profile and structural arrangement. There are several mutations known on the CXCL12 protein. One important mutation is H25R substitution at the binding interface which discourages CXCL12 dimerization, thus it is preferentially monomeric^76, 79^. However, some studies have also reported that binding of the CXCR4 N-terminus (CXCR4_NT_) induces H25R mutated CXCL12 (CXCL12_H25R_) dimerization^80^. Here we investigate the homodimerization of CXCL12_WT_ and CXCL12_H25R_, and the interaction of CXCL12 with CXCR4_N-terminus_ by using PRISM to better understand the conformational differences and their downstream effects. Figure 11a shows the pairwise superposition of CXCL12_WT_ (PDB ID: 2j7zA) conformation and its H25R mutated conformation (PDB ID: 2kolA). As can be seen from the figure they don’t align perfectly on top of each other, especially in N- and C-terminal parts. Besides, there is a significant difference in their surfaces at residue 25 as shown in Fig. 11a. Based on our clustering method, these two conformations belong to different clusters.

The PRISM webserver predicted the homodimer formation for both CXCL12_WT_, and CXCL12_H25R_. However, in case of the CXCL12_H25R_ the binding energy diminished significantly as expected. The binding energy score for CXCL12_WT_ homodimer is −101.41, while it is −17.24 for CXCL12_H25R_ homodimer. The binding residues of these homodimer structures are listed in Supplementary Table S7. Figure 11b shows the superposition of the PRISM prediction for CXCL12_WT_ homodimer and the available structure in PDB database. There is no structure in PDB database for CXCL12_H25R_ homodimer to compare with the PRISM prediction shown in Fig. 11c.

**Figure 12:**
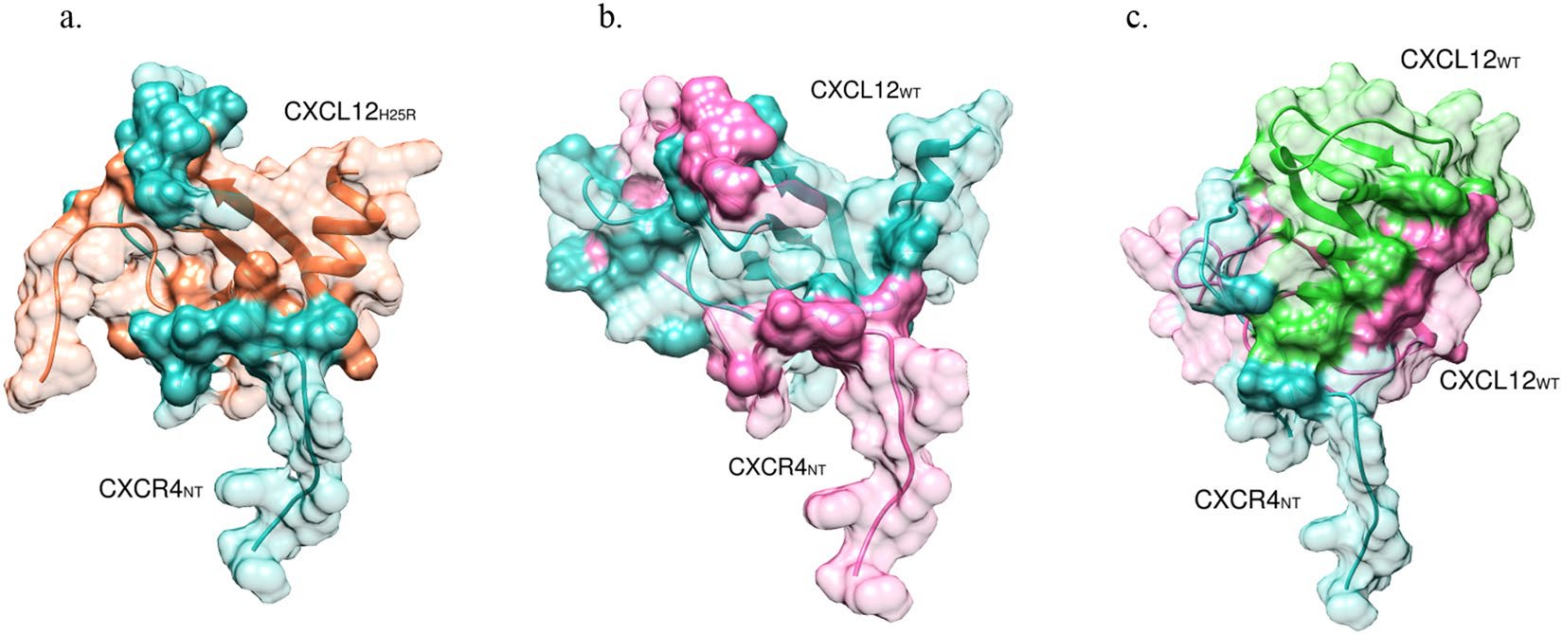
PRISM predictions for (**a**) CXCL12_H25R_ (orange)-CXCR4_NT_ (blue) interaction, (**b**) CXCL12_WT_ (pink)-CXCR4_NT_ (blue) interaction, and (**c**) CXCL12_WT_:CXCR4_NT_(pink:blue)-CXCL12_WT_ (green) trimer structure. Binding residues are indicated as opaque.

Afterwards we looked at the binding of monomeric CXCL12 variants to CXCR4_NT._ For both interactions, CXCL12_WT_-CXCR4_NT_ and CXCL12_H25R_-CXCR4_NT_, the PRISM has predictions with acceptable binding energies of −87.25 and −52.48 respectively. Tyrosine sulfation is known to promote the protein−protein interactions. There are three potential sulfation sites on CXCR4_NT_ at positions 7, 12, and 21^80^ which PRISM used two of them in its predictions (Supplementary Table S8). Figure 12a and b show the PRISM predictions for interactions CXCL12_H25R_-CXCR4_NT_ and CXCL12_WT_-CXCR4_NT_ respectively, in which CXCR4 surrounded CXCL12.

Based on these results we can conclude that CXCL12 binds to CXCR4_NT_ in wildtype and mutated forms. As monomeric CXCL12–CXCR4_NT_ interaction is said to stimulate the dimerization of CXCL12, we predicted interaction of CXCL12_WT_:CXCR4_NT_ heterodimer to CXCL12_WT_, and CXCL12_H25R_:CXCR4_NT_ heterodimer to CXCL12_H25R_. Interestingly, the PRISM could only find interaction for CXCL12_WT_:CXCR4_NT_ heterodimer to CXCL12_WT_ with the binding energy of −85.31 and could not predict a binding for CXCL12_H25R_:CXCR4_NT_ heterodimer to CXCL12_H25R_. This shows that binding of CXCR4_NT_ to CXCL12_H25R_ cannot induce homo-dimerization of CXCL12_H25R_ as it may partly occupy the dimerization interface of CXCL12_H25R_. Therefore, a single point mutation can inhibit CXCL12 dimerization which can contribute to metastatic profile and tumour dissemination. Fig. 12c shows the PRISM prediction for the trimer structure of CXCL12_WT_:CXCR4_NT_-CXCL12_WT_. Supplementary Fig. S4 and Table S9 show the superposition of this predicted complex with available dimer structure of CXCL12_WT_, and list of the binding residues of this complex structure respectively.

Consequently, as proteins are dynamic and changing conformations based on environmental factors, they favour different binding partners at each time point. Therefore, investigating alternative conformations for each protein is vital in structural PPI networks to have a precise understanding of protein interactions and signalling pathways.

We submitted all the interactions for these two case studies to Zdock^81^, GRAMM-X^82^, and PatchDock^83, 84^ webservers too. The common residues between their predictions and PRISM predictions are listed in Supplementary Tables 10 and 11.

### Mutual Exclusive Interactions of NEDD9 with SMAD3 and NCK1

**Figure 13:**
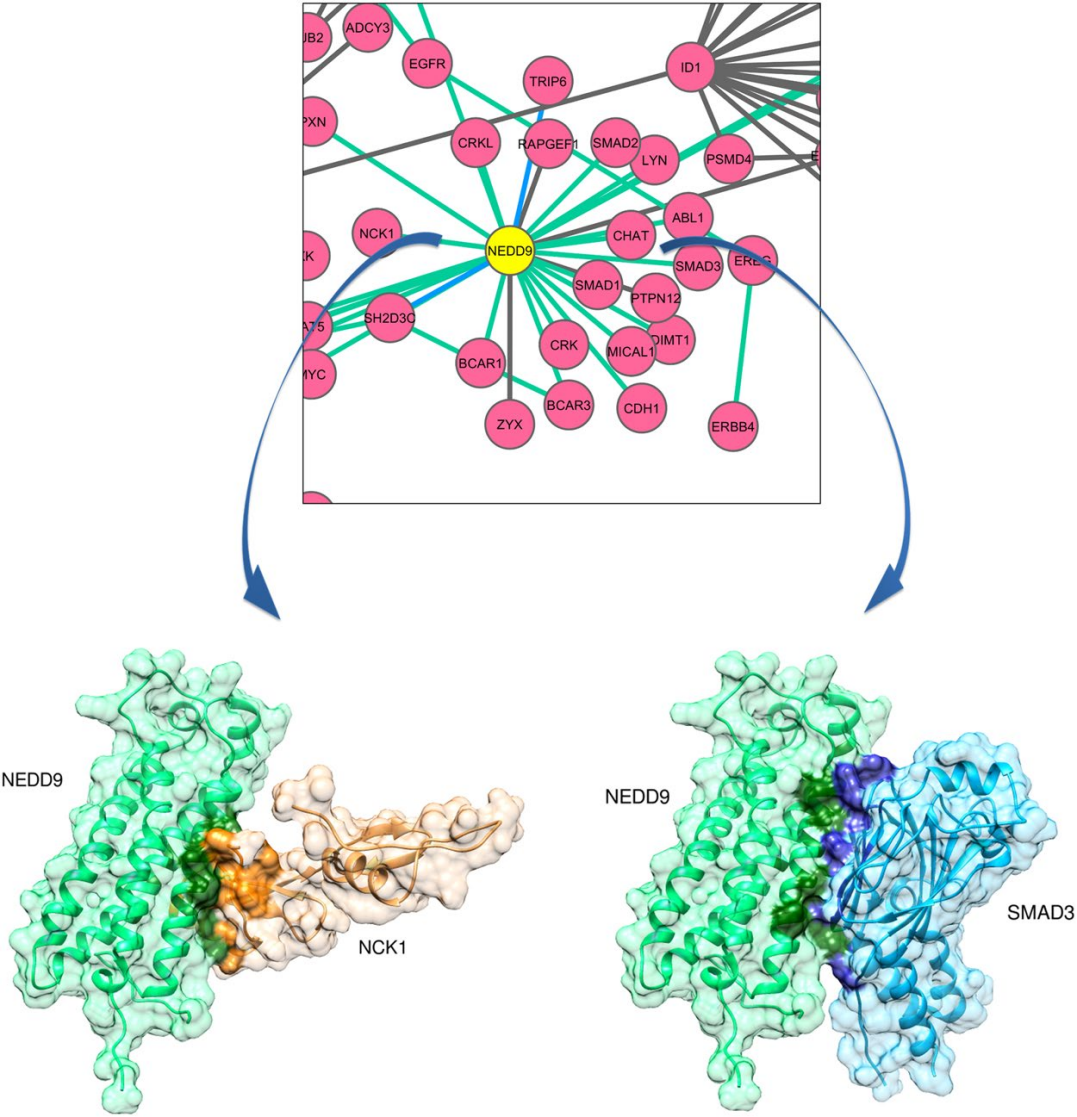
NEDD9 cluster in LMSN and PRISM predictions for its interactions with NEDD9 and SMAD3. (Top) Colour of the edges corresponds to PDB and PRISM coverages. Blue edges are covered by PDB, and green edges are covered by PRISM. (Bottom) Binding residues are indicated with opaque colours in each complex protein structure.

NEDD9 (neural precursor cell expressed, developmentally down regulated 9), SMAD3 (SMAD family member 3), and NCK1 (NCK adaptor protein 1) complex is an example of mutually exclusive interactions in LMSN. NEDD9 is a scaffolding protein, and it was previously identified as the lung metastasis causing gene in breast cancer^35^. It was used as a seed gene in LMSN creation and previously identified as metastatic hub protein in cancer signalling^85^. Figure 13 shows NEDD9 cluster i.e. its first neighbours in LMSN. NEDD9 is associated with many cellular processes such as cell adhesion, migration, proliferation, apoptosis and homeostasis^86^. The major role of NEDD9 is to promote assembly of proteins in order to maintain these cellular processes through cell signalling.

Both N-terminal and C-terminal sequences of NEDD9 are known to bind to SMAD3 causing rapid turnover of NEDD9^87, 88^. It has been found that the residues 301–330 of SMAD3 (PDB ID: 1MK2), which forms the MH2 domain, are more important for binding to NEDD9^87^. NEDD9 has only one structure available in PDB which covers residues 399–563 (PDB ID: 2L81) presenting a serine-rich four helix bundle domain. As shown in Fig. 13, the PRISM webserver predicted that NEDD9 binds to SMAD3 MH2 domain with binding energy score of −34.29.

On the other hand, PRISM predictions suggest that NEDD9 binds to NCK1 from the same region it binds to SMAD3 (Fig. 13). The binding energy score of the prediction is −22.46. NCK1 is cytoplasmic non-catalytic region of tyrosine kinase adaptor protein which facilitates the construction of larger protein assemblies. NCK1 has both SH2 and SH3 domains in its structure^89^. Previously, it was identified that NCK1 binds to NEDD9 by using its SH2 domain^90^. We observed the same result in the PRISM predictions. The predicted interface of NEDD9-NCK1 interaction consists of 26 residues and NEDD9-SMAD3 interaction interface consists of 33 residues. There are 7 residues in common between these interfaces (Supplementary Table S12).

Based on these results, NEDD9-SMAD3 and NEDD9-NCK1 interactions are mutually exclusive so we cannot see these two edges at the same time in the PPI network. Thus, to achieve to the actual topology of a PPI network we need to inspect the structural aspects of proteins. By considering the mutual exclusive interactions, the degree of NEDD9 in LMSN at any specific time point may decrease significantly.

## Conclusions

The main objective of this study is to emphasize the importance of investigating alternative conformations of proteins in structural PPI networks to be able to analyse the protein interactions more accurately. This information enables us to observe the effects of conformational changes, mutations, and mutual exclusiveness on specific protein interactions and downstream signalling.

To this end, we have built extensive protein-protein interaction network for breast cancer lung and brain metastasis by combining various databases. Then, we ranked all available interactions according to their relevance to specific breast cancer metastasis phenotypes. Using the STRING database, we filtered our network to increase the reliability of the protein interactions. To map the protein structures into the network, we proposed a clustering algorithm based on sequence and structural similarity of protein structures to prevent repetition in our analyses.

We used PRISM to model the 3D structures of the PPIs in our networks. However, we could not predict the whole interactions in our metastasis subnetworks since not all of the proteins have structures stored in the PDB database. By using alternative conformations of proteins in our subnetworks, we observed the effects of protein conformational changes on protein interactions and downstream signalling. We inspected the conformational changes of KPNB1 which directs it to bind to SNAI1 in the open conformation and bind to SNUPN in the open/close conformation. It shows the limitations posed by the conformational changes of proteins which fluctuates their ability to stablish various interactions. This is an example of conformational change effects exist in the PDB database. As another case study, we examined the interactions CXCL12_WT_-CXCR4_NT_ and CXCL12_H25R_-CXCR4_NT_. Based on our results, CXCL12_H25R_ and CXCL12_WT_ can bind to CXCR4_NT_, and this interaction has been shown to direct the migration of metastatic breast cancer cells to specific tissues. However, binding to CXCR4_NT_ inhibits CXCL12_H25R_ dimerization which is not the case for CXCL12_WT_. Dimerization of CXCL12 as the ligand of CXCR4 is important as it stimulates intracellular calcium flux but fails to activate F-actin polymerization or β-arrestin 2 recruitment which has been shown to cause cellular idling that blocks the metastatic tumour formation.

Investigating the structural models, we detected the mutual exclusive interactions. As a case study, we looked at NEDD9 mutually exclusive interaction partners. PRISM predicted that NEDD9-SMAD3 and NEDD9-NCK1 interactions are mutually exclusive. This difference can cause activation of different pathways which lead to different metastasis phenotypes. It is known that NEDD9-SMAD3 interaction causes proteolytic cleavage of NEDD9 which induces cell detachment, apoptosis and individual motility of metastasis cells. However, exact role of NCK1-NEDD9 binding was not previously examined in detail.

Overall, we created the brain and lung metastasis subnetworks of breast cancer and modelled the 3D structures of the protein-protein interactions to describe the significance of protein structural data and conformational changes through the several case studies. We believe that this study may be beneficial to appreciate the inability of traditional PPI networks, which lack structural and dynamics information of proteins, to provide a detailed view of protein interactions and their functions.

## Electronic supplementary material

Supplementary information
