# Supplementary material for: Enriching Traditional Protein-protein Interaction Networks with Alternative Conformations of Proteins
Source: Sci Rep. 2017 Aug 3;7:7180. doi: 10.1038/s41598-017-07351-0 (PMC5543104; doi:10.1038/s41598-017-07351-0)
Supplement: Supplementary file 1 — Supplementary information [file 41598_2017_7351_MOESM1_ESM.pdf]

Enriching Traditional Protein-protein Interaction Networks with Alternative Conformations of Proteins

Farideh Halakou[1], Emel Sen Kilic[2, ~], Engin Cukuroglu[3], Ozlem Keskin[2], and Attila Gursoy[1, *]

Supplementary Table S1: The genes mediating breast cancer to lung and brain metastases based on [30, 31].

**Genes associated with lung metastasis**	**Genes associated with brain metastasis**
MMP1	MMP1
RARRES3	RARRES3
FSCN1	FSCN1
ANGPTL4	ANGPTL4
LTBP1	LTBP1
PTGS2	PTGS2
KYNU	SEPP1
TNC	LAMA4
C10orf116	PLOD2
CXCL1	COL13A1
CXCR4	SCNN1A
KRTHB1 (KRT81)	RGC32
VCAM1	PELI1
LY6E	TNFSF10
EREG	B4GALT6
NEDD9	HBEGF
MAN1A1	CSF3
ID1	

Supplementary Table S2: Human interaction network extracted from STRING Database.

**STRING database protein interactions**	**Number of interactions**	**Number of proteins**
Whole human protein interactions	4,850,628	20,770
Experimentally proved human protein interactions	618,574	16,571
Experimentally proved medium confidence score human protein interactions	165,184	12,260

Supplementary Table S3: RMSD values of aligned structures of KPNB1's alternative conformations.

	**2q5dB**	**1qgkA**	**3lwwC**	**1qgrA**
**3w5kA**	3.65 Å	3.69 Å	4.33 Å	4.39 Å
**2q5dB**		3.09 Å	5.05 Å	4.12 Å
**1qgkA**			4.34 Å	2.87 Å
**3lwwC**				3.22 Å

Supplementary Table S4: Interacting residues in protein complex 3lwwCD representing the interactions KPNB1-SNUPN.

**Interface Name**	**Residue Number**	**Residue Type**	**Chain**
3LWWCD	533	MET	C
3LWWCD	534	GLU	C
3LWWCD	583	LEU	C
3LWWCD	**586**	**ALA**	**C**
3LWWCD	**589**	**GLN**	**C**
3LWWCD	**626**	**GLU**	**C**
3LWWCD	**630**	**MET**	**C**
3LWWCD	637	GLU	C
3LWWCD	**665**	**GLN**	**C**
3LWWCD	**672**	**GLY**	**C**
3LWWCD	**676**	**ASP**	**C**
3LWWCD	**679**	**ARG**	**C**
3LWWCD	682	GLN	C
3LWWCD	712	GLN	C
3LWWCD	715	SER	C
3LWWCD	**719**	**ASP**	**C**
3LWWCD	**722**	**LEU**	**C**
3LWWCD	**763**	**GLU**	**C**
3LWWCD	**770**	**THR**	**C**
3LWWCD	**774**	**GLN**	**C**
3LWWCD	**820**	**GLY**	**C**
3LWWCD	**824**	**ASP**	**C**
3LWWCD	**861**	**LEU**	**C**
3LWWCD	**864**	**TRP**	**C**
3LWWCD	43	ARG	D
3LWWCD	44	ARG	D
3LWWCD	46	ARG	D
3LWWCD	47	LEU	D
3LWWCD	48	LEU	D
3LWWCD	50	LEU	D
3LWWCD	51	GLN	D
3LWWCD	52	LYS	D
3LWWCD	54	LYS	D
3LWWCD	55	ARG	D
3LWWCD	58	TYR	D
3LWWCD	59	VAL	D
3LWWCD	62	ALA	D
3LWWCD	63	ARG	D
3LWWCD	64	ARG	D

Bold residues show the common binding residues of KPNB1 to interact with SNUPN and KPNA2.

Supplementary Table S5: Interacting residues in protein complexes 1qgkAB representing the interaction KPNB1-KPNA2.

**Interface Name**	**Residue Number**	**Residue Type**	**Chain**
1QGKAB	*281*	*GLU*	*A*
1QGKAB	*284*	*SER*	*A*
1QGKAB	*285*	*ASN*	*A*
1QGKAB	*288*	*ASP*	*A*
1QGKAB	339	ASP	A
1QGKAB	*340*	*ASP*	*A*
1QGKAB	*342*	*TRP*	*A*
1QGKAB	346	LYS	A
1QGKAB	*350*	*VAL*	*A*
1QGKAB	*353*	*MET*	*A*
1QGKAB	*354*	*LEU*	*A*
1QGKAB	*388*	*MET*	*A*
1QGKAB	*426*	*ASP*	*A*
1QGKAB	*427*	*THR*	*A*
1QGKAB	*430*	*TRP*	*A*
1QGKAB	469	ASN	A
1QGKAB	*472*	*TRP*	*A*
1QGKAB	*530*	*GLU*	*A*
1QGKAB	*582*	*SER*	*A*
1QGKAB	**586**	**ALA**	**A**
1QGKAB	**589**	**GLN**	**A**
1QGKAB	593	ARG	A
1QGKAB	*623*	*GLY*	*A*
1QGKAB	***626***	***GLU***	***A***
1QGKAB	*627*	*ASP*	*A*
1QGKAB	**630**	**MET**	**A**
1QGKAB	**665**	**GLN**	**A**
1QGKAB	**672**	**GLY**	**A**
1QGKAB	**676**	**ASP**	**A**
1QGKAB	**679**	**ARG**	**A**
1QGKAB	**719**	**ASP**	**A**
1QGKAB	**722**	**LEU**	**A**
1QGKAB	**763**	**GLU**	**A**
1QGKAB	767	GLU	A
1QGKAB	**770**	**THR**	**A**
1QGKAB	**774**	**GLN**	**A**
1QGKAB	**820**	**GLY**	**A**
1QGKAB	**824**	**ASP**	**A**
1QGKAB	860	THR	A

1QGKAB	**861**	**LEU**	**A**
1QGKAB	**864**	**TRP**	**A**
1QGKAB	11	ALA	B
1QGKAB	12	ALA	B
1QGKAB	13	ARG	B
1QGKAB	14	LEU	B
1QGKAB	16	ARG	B
1QGKAB	17	PHE	B
1QGKAB	18	LYS	B
1QGKAB	19	ASN	B
1QGKAB	20	LYS	B
1QGKAB	22	LYS	B
1QGKAB	27	MET	B
1QGKAB	28	ARG	B
1QGKAB	31	ARG	B
1QGKAB	32	ILE	B
1QGKAB	34	VAL	B
1QGKAB	35	ASN	B
1QGKAB	38	LEU	B
1QGKAB	39	ARG	B
1QGKAB	40	LYS	B
1QGKAB	43	LYS	B
1QGKAB	47	MET	B
1QGKAB	50	ARG	B
1QGKAB	51	ARG	B
1QGKAB	53	VAL	B
1QGKAB	54	SER	B

Bold and italic residues show the common binding residues of KPNB1 to interact with KPNA2 and SNUPN, and to interact with KPNA2 and SNAI1 respectively.

Supplementary Table S6: Interacting residues in protein complexes 3w5kAB representing the interaction KPNB1-SNAI1.

**Interface Name**	**Residue Number**	**Residue Type**	**Chain**
3W5KAB	200	ASN	A
3W5KAB	242	VAL	A
3W5KAB	243	LYS	A
3W5KAB	246	SER	A
3W5KAB	247	LEU	A
3W5KAB	*281*	*GLU*	*A*
3W5KAB	*284*	*SER*	*A*
3W5KAB	*285*	*ASN*	*A*
3W5KAB	*288*	*ASP*	*A*
3W5KAB	289	GLU	A
3W5KAB	292	ASP	A
3W5KAB	*340*	*ASP*	*A*
3W5KAB	*342*	*TRP*	*A*
3W5KAB	*350*	*VAL*	*A*
3W5KAB	*353*	*MET*	*A*
3W5KAB	*354*	*LEU*	*A*
3W5KAB	*388*	*MET*	*A*
3W5KAB	395	GLU	A
3W5KAB	399	PRO	A
3W5KAB	*426*	*ASP*	*A*
3W5KAB	*427*	*THR*	*A*
3W5KAB	*430*	*TRP*	*A*
3W5KAB	437	GLU	A
3W5KAB	438	LEU	A
3W5KAB	440	PRO	A
3W5KAB	441	GLU	A
3W5KAB	*472*	*TRP*	*A*
3W5KAB	479	GLU	A
3W5KAB	480	ALA	A
3W5KAB	483	GLU	A
3W5KAB	491	GLN	A
3W5KAB	*530*	*GLU*	*A*
3W5KAB	572	SER	A
3W5KAB	575	ILE	A
3W5KAB	576	GLN	A
3W5KAB	578	ASN	A
3W5KAB	579	ASP	A
3W5KAB	*582*	*SER*	*A*
3W5KAB	583	LEU	A

3W5KAB	621	SER	A
3W5KAB	622	GLY	A
3W5KAB	*623*	*GLY*	*A*
3W5KAB	624	VAL	A
3W5KAB	*626*	*GLU*	*A*
3W5KAB	*627*	*ASP*	*A*
3W5KAB	157	LYS	B
3W5KAB	165	SER	B
3W5KAB	166	LEU	B
3W5KAB	167	GLY	B
3W5KAB	170	LYS	B
3W5KAB	173	ILE	B
3W5KAB	191	ARG	B
3W5KAB	192	PRO	B
3W5KAB	193	TRP	B
3W5KAB	196	GLN	B
3W5KAB	200	ARG	B
3W5KAB	220	ARG	B
3W5KAB	221	SER	B
3W5KAB	222	ASN	B
3W5KAB	224	ARG	B
3W5KAB	225	ALA	B
3W5KAB	228	GLN	B
3W5KAB	229	THR	B
3W5KAB	234	LYS	B
3W5KAB	237	GLN	B
3W5KAB	239	GLN	B
3W5KAB	240	ALA	B
3W5KAB	241	CYS	B
3W5KAB	242	ALA	B
3W5KAB	243	ARG	B
3W5KAB	246	SER	B
3W5KAB	247	ARG	B
3W5KAB	249	SER	B
3W5KAB	250	LEU	B
3W5KAB	253	LYS	B
3W5KAB	261	GLY	B
3W5KAB	262	CYS	B
3W5KAB	263	PRO	B
3W5KAB	264	ARG	B

Italic residues show the common binding residues of KPNB1 to interact with SNAI1 and KPNA2.

Supplementary Table S7: Predicted binding residues of $CXCL12_{WT}$ (PDB ID: 2j7zA) homodimer and $CXCL12_{H25R}$ (PDB ID: 2kolA) homodimer.

**2j7zA**		**2j7zA**	
ILE	28	TYR	61
ILE	28	LEU	62
LEU	29	TYR	61
ILE	28	ALA	65
LYS	68	ALA	35
LYS	27	LEU	26
ASN	30	VAL	23
LYS	24	ASN	30
LEU	29	HIS	25
ILE	38	LEU	66
ILE	38	ALA	65
ALA	65	GLN	37
ASN	30	TYR	61
ILE	28	ILE	28
ILE	28	HIS	25
ILE	28	LEU	26
TYR	61	LEU	29
TYR	61	ILE	28
ALA	65	ILE	38
GLN	37	ALA	65
HIS	25	LYS	27
LEU	62	LEU	62
ALA	65	PRO	53
ASN	30	LYS	24
HIS	25	LEU	29
HIS	25	ILE	28
ASN	30	ARG	20
ARG	20	ASN	30
LEU	62	ILE	28
ILE	58	LEU	66
LYS	27	LYS	27
LYS	24	LEU	29
LYS	27	HIS	25
LEU	26	LEU	26
LEU	26	LYS	27
GLN	37	LYS	68
LEU	26	ILE	28
LYS	68	CYS	34
LEU	66	ILE	38
PRO	32	LYS	68
VAL	23	ASN	30
PRO	53	LEU	66
PRO	53	ALA	65
ALA	35	LYS	68
LEU	66	ILE	58
LEU	66	PRO	53
CYS	34	LYS	68
GLN	37	LYS	64
HIS	25	ASN	30
TYR	61	ASN	30

**2kolA**		**2kolA**	
ASN	67	GLU	63
LEU	29	ARG	25
LEU	26	ASN	30
LEU	29	LYS	27
LEU	29	LEU	26
ASN	30	LEU	62
ILE	28	ILE	28
ASN	33	ARG	25
ILE	28	LYS	27
ASN	30	LEU	26
ASN	30	ARG	25
ARG	25	LEU	29
THR	31	ARG	25
GLU	63	GLU	63
LYS	68	GLU	63
GLU	63	ASN	67
GLN	59	ASN	67
LYS	27	LYS	27
LEU	36	LEU	66
LYS	68	GLN	59
LEU	26	LEU	29
LYS	27	LEU	29
LYS	27	ILE	28
LEU	66	ASN	30
LEU	66	ALA	35
LEU	66	LEU	36
PRO	10	ARG	25
ASN	67	GLN	59
PRO	32	ARG	25
PRO	32	LYS	24
ARG	8	ARG	25
ARG	25	ASN	30
LEU	62	ASN	30

Supplementary Table S8: Predicted binding residues of CXCL12$_{WT}$ (PDB ID: 2j7zA)-CXCR4$_{NT}$ (PDB ID: 2k03B), and CXCL12$_{H25R}$ (PDB ID: 2kolA)-CXCR4$_{NT}$ (2k03B).

2j7zA		2k03B	
**GLN**	**48**	**TYR**	**112**
LEU	5	THR	113
PHE	14	SER	118
PHE	14	GLY	119
GLN	59	PHE	129
LYS	56	PHE	129
LYS	54	PHE	129
ARG	8	GLU	115
LYS	54	GLU	126
THR	31	ILE	106
**THR**	**31**	**TYR**	**107**
LYS	1	GLU	115
**LEU**	**29**	**TYR**	**112**
VAL	49	SER	118
PHE	14	MET	124
PHE	14	SER	123
CYS	9	GLU	114
PHE	14	ASP	120
PRO	32	SER	105
PRO	32	ILE	104
PRO	32	ILE	106
LYS	54	PRO	127
GLU	15	SER	118
PRO	32	GLU	102
PRO	10	GLU	114
LYS	54	ALA	128
PRO	10	THR	113
ASN	33	GLY	103
**LYS**	**27**	**TYR**	**112**
ASN	33	ILE	106
ASN	33	ILE	104
SER	6	GLU	115
VAL	3	GLU	115
VAL	3	GLU	114
VAL	3	THR	113
**VAL**	**39**	**TYR**	**112**
**ASN**	**30**	**TYR**	**107**
ASN	30	ILE	106
PHE	13	GLY	119
PHE	13	SER	118
SER	4	THR	113

2kolA		2k03B	
ASP	52	LYS	125
ASP	52	GLU	126
GLN	48	SER	118
TYR	7	GLU	115
TYR	7	GLU	114
PHE	13	SER	123
**LEU**	**29**	**TYR**	**112**
VAL	49	SER	118
VAL	49	GLY	119
PHE	14	LYS	125
PHE	14	MET	124
PHE	14	SER	123
PRO	32	SER	105
PRO	32	ILE	104
**PRO**	**32**	**TYR**	**107**
PRO	32	ILE	106
LYS	54	PRO	127
PRO	32	GLY	103
PRO	32	GLU	102
LYS	54	PHE	129
LYS	54	ALA	128
ASN	33	GLY	103
ASN	33	ILE	106
ASN	33	ILE	104
CYS	50	SER	118
CYS	50	GLY	119
LYS	56	PHE	129
PHE	13	GLY	119

Bold rows represent the interactions of three sulfation sites on CXCR4$_{NT}$ at positions 7, 12, and 21.

Supplementary Table S9: Predicted binding residues of $CXCL12_{WT}$:$CXCR4_{NT}$-$CXCL12_{WT}$ trimer structure.

**$CXL12_{WT}$:$CXCR4_{NT}$**		**CXCL12**	
ILE	28	TYR	61
ILE	28	LEU	62
LEU	29	TYR	61
LYS	68	ALA	35
MET	101	LYS	68
ASN	30	VAL	23
LYS	24	ASN	30
LEU	29	HIS	25
ILE	38	LEU	66
ILE	38	ALA	65
ALA	65	GLN	37
ASN	30	TYR	61
ILE	28	ILE	28
ILE	28	HIS	25
ASN	30	LYS	43
LEU	66	ILE	58
TYR	61	LEU	29
ILE	28	LEU	26
ALA	65	ILE	38
LEU	62	LEU	66
HIS	25	LYS	27
LEU	62	LEU	62
ALA	65	PRO	53
ASN	30	LYS	24
HIS	25	LEU	29
HIS	25	ILE	28
TYR	61	ILE	28
ARG	20	ASN	30
GLU	63	GLU	63
LEU	62	ILE	28
ILE	58	LEU	66
GLN	59	LEU	66
LYS	27	LEU	26
LEU	26	LEU	26
LEU	26	LYS	27
TYR	107	LYS	24
LEU	26	ILE	28
LEU	66	ILE	38
LYS	54	ASN	67

LYS	54	LEU	66
LYS	54	LYS	68
VAL	23	ASN	30
PRO	127	LYS	68
PRO	53	LEU	66
GLU	126	LYS	68
LEU	66	GLN	59
PRO	53	ALA	65
ASN	67	PRO	53
TYR	61	ASN	30
LEU	66	LYS	54
LEU	66	PRO	53
LYS	68	GLN	37
HIS	25	ASN	30

Supplementary Table S10: Common interface residues in PRISM and other docking methods predictions for interactions of KPNB1 with SNUPN and KPNA2.

**Docking Tools**	**Common Interface Residues in Interaction**
	**KPNB1-SNUPN (3w5kA-3lwwD)**
**PRISM and PatchDock**	A:630,A:637,A:672,A:676,A:679,A:712,A:715,A:719,A:722,A:767,A:770,A:774,A:820,A:824,A:857,A:861, A:864,D:43,D:44,D:47,D:50,D:51,D:54,D:55,D:58,D:59,D:62,D:63
**PRISM and GRAMM-X**	A:665,D:50,D:56,D:63
**PRISM and Zdock**	A:626,A:630,A:637,A:665,A:672,A:676,A:679,A:712,A:715,A:719,A:722,A:767,A:770,A:774,A:820,A:824, A:857,A:861,A:864,D:44,D:47,D:48,D:50,D:51,D:54,D:55,D:56,D:58,D:59,D:62,D:63,D:64
	**KPNB1-SNUPN (2q5dB-3lwwD)**
**PRISM and PatchDock**	B:630,B:633,B:665,B:676,B:712,D:40,D:43,D:55,D:59,D:62,D:63,D:64
**PRISM and GRAMM-X**	B:857,D:40,D:43,D:44,D:62,D:63,D:64
**PRISM and Zdock**	B:589,B:593,B:679,D:43,D:44,D:47,D:51,D:55,D:59,D:62,D:63,D:64
	**KPNB1-SNUPN (1qgkA-3lwwD)**
**PRISM and PatchDock**	A:586,A:589,A:627,A:630,A:665,A:668,A:672,A:679,A:712,A:715,D:43,D:44,D:47,D:50,D:51,D:54,D:55,D:58, D:59,D:62,D:63
**PRISM and GRAMM-X**	A:679,A:682,A:719,A:722,A:723,A:770,A:774,A:824,D:51,D:55,D:58,D:59,D:62,D:63
**PRISM and Zdock**	A:593,A:630,A:633,A:637,A:665,A:672,A:676,A:679,A:712,D:43,D:44,D:47,D:50,D:54,D:55,D:58,D:59,D:62, D:63
	**KPNB1-SNUPN (1qgrA-3lwwD)**

**PRISM and PatchDock**	A:586,A:589,A:593,A:627,A:630,A:637,A:676,A:679,A:715,A:722,A:767,A:770,A:774,A:777,A:779,A:820,A:824, A:827,A:861,A:864,D:43,D:47,D:50,D:51,D:54,D:55,D:56,D:58,D:59,D:61,D:62,D:63,D:64
**PRISM and GRAMM-X**	A:679,A:682,A:719,A:722,A:767,A:770,A:774,A:779,A:820,A:824,A:861,A:864,D:51,D:52,D:55,D:56,D:58,D:59, D:61,D:62,D:63,D:64
**PRISM and Zdock**	A:626,A:630,A:637,A:665,A:672,A:676,A:679,A:682,A:715,A:719,A:722,A:763,A:767,A:770,A:774,A:779,A:824,A:861, A:864,D:43,D:44,D:47,D:51,D:52,D:54,D:55,D:56,D:58,D:59,D:62,D:63,D:64
	**KPNB1-KPNA2 (3lwwC-1qgkB)**
**PRISM and PatchDock**	B:11,B:12,B:13,B:16,B:28,B:36,B:39,B:40,B:42,B:44,B:51,B:53,B:54,C:340,C:589,C:630,C:679,C:719,C:767
**PRISM and GRAMM-X**	B:11,B:12,B:14,B:19,B:20,B:31,B:36,B:39,B:40,B:43,B:53,B:54,C:346,C:350,C:388,C:430
**PRISM and Zdock**	B:11,B:12,B:13,B:14,B:16,B:17,B:18,B:19,B:20,B:28,B:31,B:39,B:40,B:47,B:50,C:288,C:339,C:340,C:342,C:346,C:350, C:388,C:427,C:430,C:589,C:630,C:679,C:779,C:861
	**KPNB1-KPNA2 (3w5kA-1qgkB)**
**PRISM and PatchDock**	A:350,A:434,A:582,A:626,A:630,A:665,A:668,A:672,A:711,A:712,A:715,A:767,B:11,B:12,B:13,B:14,B:20,B:23,B:24,B:25, B:28,B:39,B:52
**PRISM and GRAMM-X**	B:11,B:12,B:13,B:14,B:23,B:25,B:51,B:52
**PRISM and Zdock**	A:284,A:288,A:340,A:342,A:350,A:354,A:388,A:430,A:469,A:575,A:576,A:579,A:855,B:11,B:12,B:13,B:14,B:17,B:18,B:19, B:24,B:25,B:28,B:31,B:50,B:51,B:52
	**KPNB1-KPNA2 (2q5dB-1qgkB)**
**PRISM and PatchDock**	A:593,A:679,A:722,B:12,B:13,B:14,B:16,B:17,B:18,B:19,B:20,B:22,B:23,B:24,B:27,B:28,B:31,B:35,B:38,B:42,B:46,B:48, B:50,B:51,B:53,B:54
**PRISM and GRAMM-X**	A:593,A:679,B:16,B:18,B:19,B:22,B:24,B:27,B:28,B:31,B:35,B:48,B:51,B:53,B:54
**PRISM and Zdock**	A:340,A:341,A:342,A:346,A:349,A:350,A:353,A:388,A:430,A:472,A:526,A:530,A:533,A:583,A:593,A:679,A:722,A:770, A:857,A:864,B:12,B:13,B:14,B:16,B:17,B:18,B:19,B:20,B:22,B:24,B:27,B:28,B:31,B:35,B:43,B:48,B:51,B:53,B:54
	**KPNB1-KPNA2 (1qgrA-1qgkB)**
**PRISM and PatchDock**	A:626,A:672,A:676,A:679,A:682,A:719,A:763,B:11,B:12,B:13,B:16,B:28,B:39,B:40,B:51,B:53
**PRISM and GRAMM-X**	B:28,B:39,B:42,B:46,B:52,B:53

**PRISM and Zdock**	A:626,A:672,A:676,A:679,A:682,A:719,A:763,B:11,B:12,B:13,B:16,B:28,B:39,B:40,B:51,B:53

Supplementary Table S11: Common interface residues in PRISM and other docking methods predictions for $CXCL12_{WT}$ and $CXCL12_{H25R}$ homodimers and their interactions with $CXCR4_{NT}$.

**Docking Tools**	**Common Interface Residues for interaction**
	**$CXCL12_{WT}$ homodimer (2j7zA-2j7zA)**
**PRISM and PatchDock**	A:23,A:24,A:25,A:26,A:27,A:28,A:29,A:30,A:35,A:37,A:38,A:53,A:58,A:61,A:62,A:65,A:66,A:68,B:23,B:24,B:25,B:26, B:27,B:28,B:29,B:30,B:35,B:37,B:38,B:53,B:58,B:61,B:62,B:65,B:66,B:68
**PRISM and GRAMM-X**	A:23,A:24,A:25,A:26,A:27,A:28,A:29,A:30,A:35,A:37,A:38,A:53,A:58,A:61,A:62,A:65,A:66,A:68,B:23,B:24,B:25,B:26, B:27,B:28,B:29,B:30,B:35,B:37,B:38,B:53,B:58,B:61,B:62,B:65,B:66,B:68
**PRISM and Zdock**	A:34,A:35,A:53,B:32,B:34,B:35,B:53
	**$CXCL12_{H25R}$ homodimer (2kolA-2kolA)**
**PRISM and PatchDock**	A:25,A:27,A:29,A:30,B:10,B:25,B:27,B:29,B:30,B:31,B:32
**PRISM and GRAMM-X**	No common residues.
**PRISM and Zdock**	No common residues.
	**$CXCL12_{WT}$-$CXCR4_{NT}$ (2j7zA-2k03B)**
**PRISM and PatchDock**	A:1,A:4,A:5,A:6,A:8,A:13,A:14,A:32,A:54,A:56,A:59,B:106,B:107,B:112,B:113,B:114,B:118,B:120,B:123,B:124,B:126, B:127,B:128,B:129
**PRISM and GRAMM-X**	A:1,A:3,A:10,A:13,A:27,A:29,A:30,B:102,B:103,B:106,B:112,B:118,B:119,B:120,B:123,B:124,B:126,B:127,B:128,B:129
**PRISM and Zdock**	A:1,A:4,A:10,A:13,A:27,A:29,A:30,B:102,B:103,B:114,B:119,B:120,B:123,B:124,B:126,B:127
	**$CXCL12_{H25R}$-$CXCR4_{NT}$ (2kolA-2k03B)**
**PRISM and PatchDock**	A:7,A:13,A:32,A:33,A:49,A:54,A:56,B:102,B:103,B:104,B:105,B:106,B:107,B:112,B:114,B:118,B:119,B:124,B:125,B:126, B:128,B:129

**PRISM and GRAMM-X**	A:7,A:13,A:14,A:29,A:32,A:48,A:49,A:52,A:54,A:56,B:103,B:104,B:106,B:107,B:112,B:114,B:115,B:118,B:119,B:123, B:124,B:125,B:126,B:127,B:128,B:129
**PRISM and Zdock**	A:7,A:14,A:32,A:33,A:49, A:56,B:102,B:103,B:104,B:106,B:107,B:112,B:114, B:119,B:124,B:125,B:126, B:128
	**CXCL12$_{wt}$:CXCR4NT- CXCL12$_{wt}$ (2j7zA:2k03B-2j7zA)**
**PRISM and PatchDock**	A:24,A:25,A:27,A:29,A:30,C:24,C:25,C:27,C:29,C:30
**PRISM and GRAMM-X**	A:24,A:25,A:26,A:27,A:28,A:30,A:54,A:59,A:61,A:62,A:65,A:66,A:68,C:24,C:25,C:26,C:27,C:28,C:30,C:37,C:54,C:59, C:61,C:62,C:65,C:66,C:68
**PRISM and Zdock**	A:53,A:54,A:59,C:35,C:53,C:54,C:59

Supplementary Table S12: Predicted binding residues of NEDD9 (PDB ID: 2l81A)-SMAD3 (PDB ID: 1mk2A), and NEDD9 (PDB ID: 2l81A)-NCK1 (2ci8A) interactions.

**1mk2A**		**2l81A**	
SER	253	**ALA**	**32**
SER	253	**GLN**	**31**
SER	253	**MET**	**35**
PRO	335	LEU	43
PRO	252	**ARG**	**28**
PRO	252	GLN	27
LEU	307	**SER**	**38**
TYR	296	**ALA**	**32**
TYR	297	**ARG**	**28**
CYS	337	GLU	60
PHE	303	**MET**	**35**
GLY	336	LEU	43
TYR	296	**MET**	**35**
TYR	296	**ARG**	**28**
GLY	336	ALA	64
ASP	309	ALA	42
LEU	307	ALA	42
ARG	291	ALA	42
PRO	335	HIS	57
ARG	294	**MET**	**35**
PRO	252	**GLN**	**31**
GLU	227	**ARG**	**28**
ASN	338	**LYS**	**67**
LEU	295	**MET**	**35**
GLU	305	**PHE**	**71**
PRO	335	GLU	60
ARG	291	MET	41
ARG	294	**SER**	**38**
GLU	305	SER	39
GLU	305	GLY	36
LEU	307	SER	39
ILE	298	**ARG**	**28**
GLU	305	**MET**	**35**
GLY	336	SER	39

**2l81A**		**2ci8A**	
**ARG**	**28**	TYR	284
**ARG**	**28**	VAL	287
LEU	70	HIS	301
LEU	70	GLY	300
LEU	70	ASP	304
HIS	77	ARG	299
GLU	74	GLY	300
**PHE**	**71**	ASP	304
**PHE**	**71**	PHE	305
LEU	29	ARG	299
**MET**	**35**	TRP	282
**MET**	**35**	PRO	281
PHE	78	ARG	299
GLU	34	PRO	281
**GLN**	**31**	TYR	284
**GLN**	**31**	TYR	283
ARG	28	GLN	291
**MET**	**35**	LEU	306
**LYS**	**67**	PHE	305
MET	35	ILE	307
**MET**	**35**	PHE	305
**ALA**	**32**	TYR	284
**SER**	**38**	PRO	281
**SER**	**38**	TRP	282
LEU	70	ARG	299
GLU	74	ARG	299

Bold residues show the common binding residues of NEDD9 to interact with SMAD3 and NCK1.

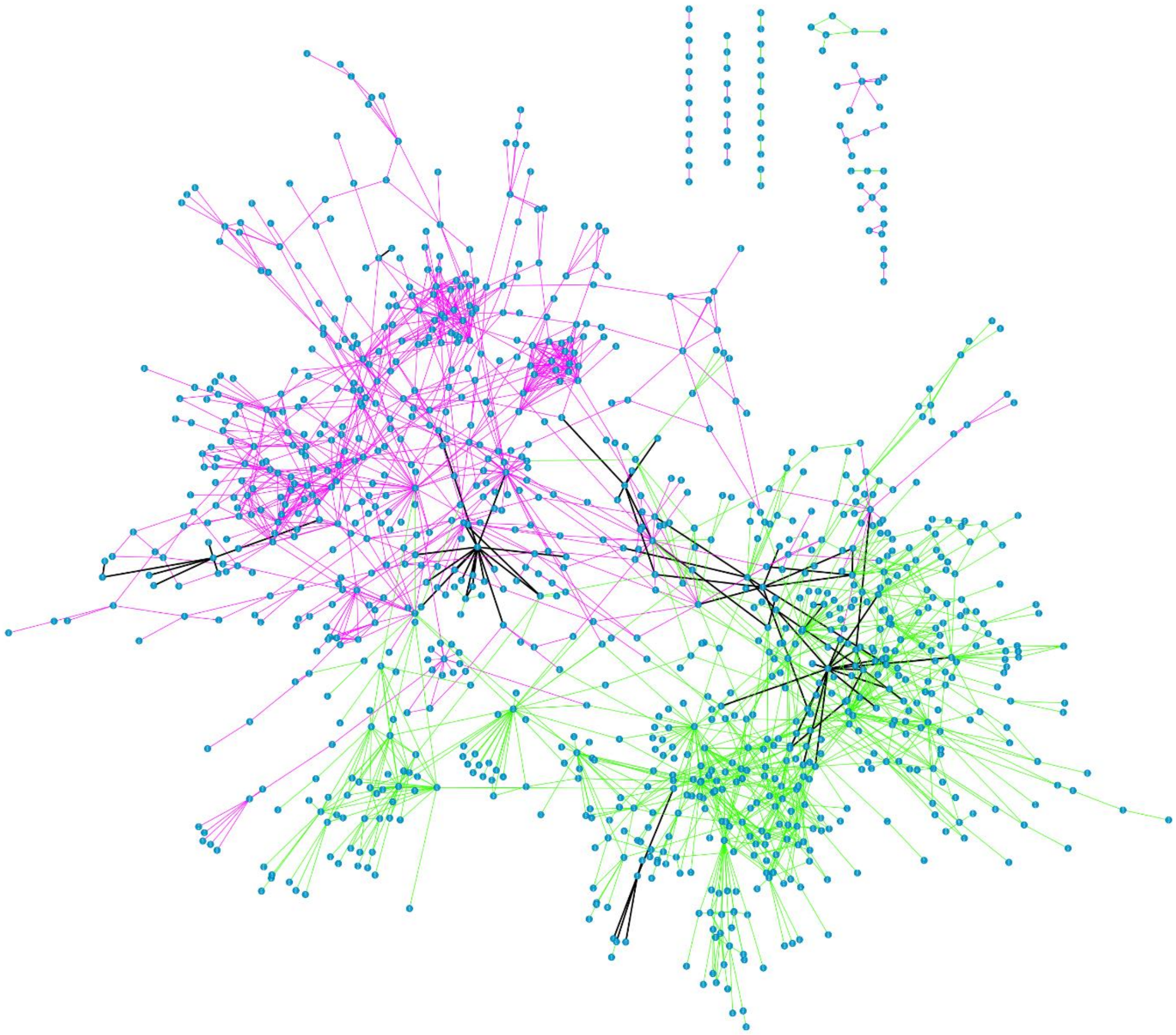

Supplementary Figure S1: The union of BMSN and LMSN top 1000 edges network. Purple, green and black edges indicate BMSN, LMSN, and common edges between two subnetworks respectively.

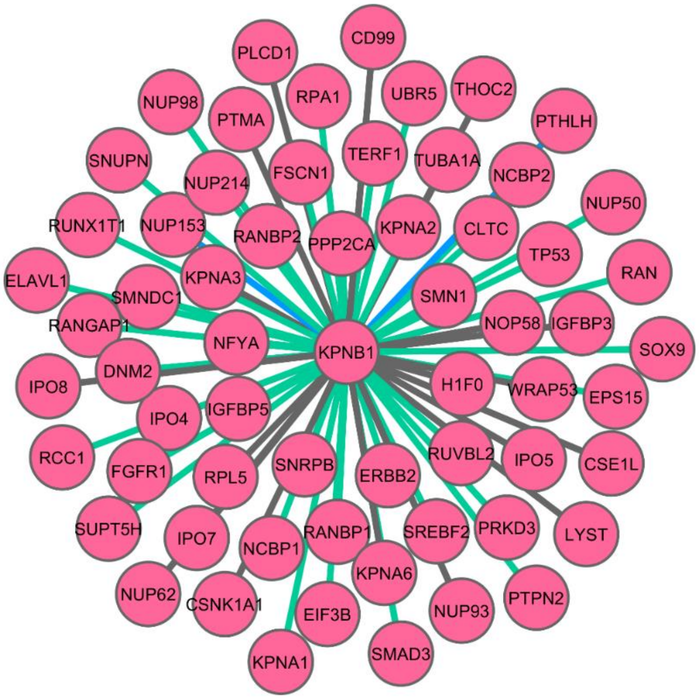

Supplementary Figure S2: KPNB1 cluster in our network. Colour of the edges corresponds to PDB and PRISM coverages. Blue edges are covered by PDB, and green edges are covered by PRISM.

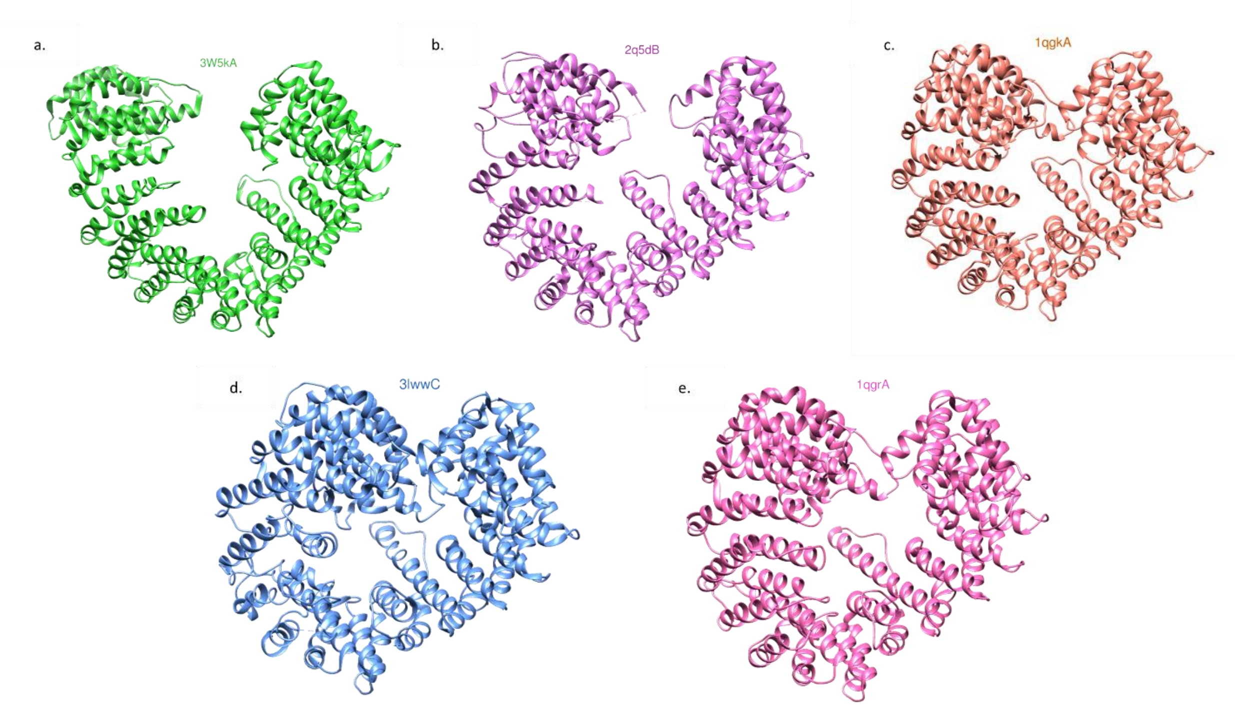

Supplementary Figure S3: Alternative conformations of KPNB1 protein. a) 3w5kA, b) 2q5dB, c) 1qgkA, d) 3lwwC, and d) 1qgrA. The RMSD value of aligned structures is shown in Supplementary Table S3.

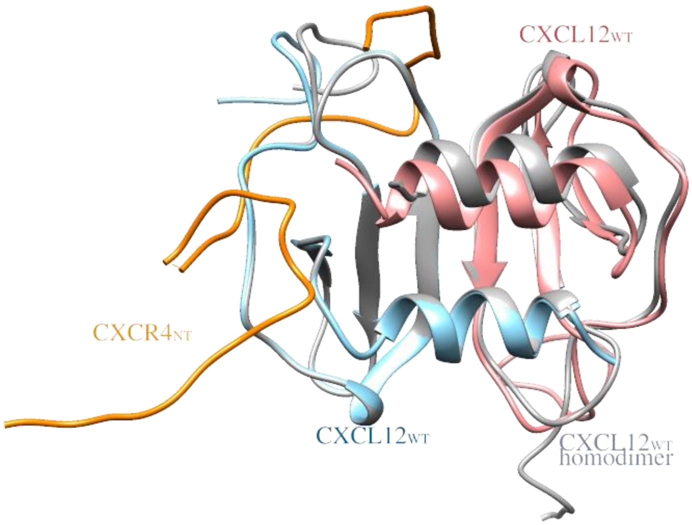

Supplementary Figure S4: Superposition of PRISM prediction for $CXCL12_{WT}$:$CXCR4_{NT}$ (blue:orange)-$CXCL12_{WT}$ (pink) trimer structure and the available $CXCL12_{WT}$ homodimer (grey) structure in PDB i.e. 2j7zAB.
